# Multi-isotope variation reveals social complexity in Viking Age Norway

**DOI:** 10.1016/j.isci.2022.105225

**Published:** 2022-09-29

**Authors:** Lisa Mariann Strand, Sam Leggett, Birgitte Skar

**Affiliations:** 1 NTNU University Museum, Department of Archaeology and Cultural History, The Norwegian University of Science and Technology, 7491 Trondheim, Norway; 2 School of History, Classics & Archaeology, The University of Edinburgh, William Robertson Wing, Old Medical School, Teviot Pl, Edinburgh EH8 9AG, UK; 3 Department of Archaeology, University of Cambridge, Downing Street, Cambridge CB2 3DZ, UK

**Keywords:** Biological sciences, evolutionary biology, archeology

## Abstract

Multi-isotope studies from human remains from Viking Age graves throughout Norway allow for a deeper understanding of mobility, livelihood, and social organization during the Viking Age (750–1050 CE). Based on a framework of radiocarbon dates (^14^C), the studied inhumation graves are distributed across a broad chronological and geographical scope, covering the Late Iron and Viking Age (c. 500–1050 CE). Results of multi-isotope analyses (δ^18^O/δ^13^C/δ^15^N) in tandem with a cultural historical approach question the hegemonic masculinity associated with the “violent Vikings” and the apparent preconception of stationary women and mobile males in Viking Age Norway, thus challenging conjectural behavioral distinctions between women, men, and children. The analysis points towards diversity following a north-south gradient in terms of dietary preferences (δ^13^C/δ^15^N), which demonstrates a higher degree of marine consumption in northern Norway, as opposed to the southern regions; similar patterns are also observed through the mobility study (δ^18^O), which uncovers high levels of migration in the study population.

## Highlights

• Diet analysis shows regional but not sex-bases differences
• Predictive origin models illustrate potential regions of childhood origins
• Critical bioarchaeological interpretations support a re-assessment of gender roles
• Our study corroborates women and children partaking in Viking Age raids and battles

## Graphical abstract

**Figure:**
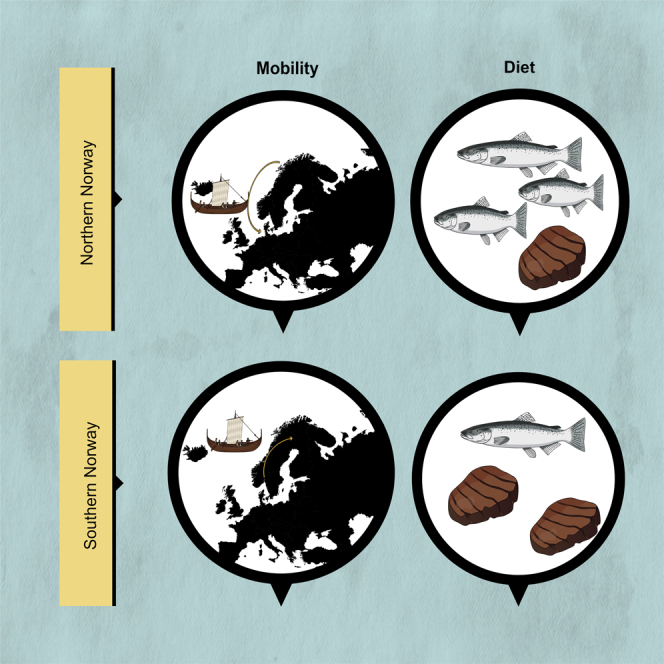

## Introduction

While often framed as hypermobile by nature, the Vikings relied on already established networks and must be seen as part of a larger northern European history of interaction (Margaryan et al., 2020). Based on written and archaeological sources, linguistics, and genetics, our view of the Viking Age (c. 750–1050 CE) is characterized by large-scale maritime movements of people from Scandinavia to Russia, the Baltic, mainland Europe, and Britain, the Atlantic islands, and Newfoundland for the purposes of trade, settling, and/or outright warfare. A recent large-scale study of Viking Age population genetics (700–1100 CE), which included the same Norwegian human individuals analyzed in the present study, revealed migratory patterns that largely confirm the thesis that ancestors with genetic affinity to the present-day population of Norway (“Norwegian-like” Vikings (Margaryan et al., 2020)) settled in Ireland, Iceland, Greenland and the Isle of Man; “Swedish-like” Vikings traveled east, and “Danish-like” Vikings traveled west (for methodological approaches see Margaryan et al., 2020: 392 and 394, Figure 4) (Margaryan et al., 2020). Another recent mitochondrial DNA study (Krzewińska et al., 2015) of human remains from the Norwegian Viking Age suggests a genetic admixture involving Norse women on the North Atlantic islands during this period. Collectively, these two studies give an overview of the migratory patterns during this period seen through the lens of genetics.

The plethora of written sources that chronicle Scandinavian Norse diasporic activity describes early small-scale raids with later periods of overwintering, more permanent encampments and, finally, settlements, all of which constitute an important backdrop for the present study. In the Insular world (Britain and Ireland), the first documented Viking incursions were between 786 and 802 CE in Portland, in Dorset, and in 793 CE at Lindisfarne, off the Northumberland coast (Dumwille, 2008). Contemporaneous raids in Ireland (795 CE) developed into seasonal campaigns that necessitated the establishment of winter encampments (Sheehan, 2008). Similar overwintering camps have been found throughout the Insular world and provide important evidence for Viking activity in our period of study (Hadley and Richards, 2016; Heen-Pettersen, 2019; Jarman et al., 2018).

The 9^th^ century onwards also saw intensified Viking activity in Continental Europe (Christys, 2015; Cooijmans, 2020). The last known Viking attack on Tiel, located centrally in what is now the Netherlands, was at the beginning of the 11^th^ century (Verwers, 2010). Despite documentary evidence for continental overwintering camps, few have been identified through archaeological research (Cooijmans, 2020). Utilizing river systems such as the Seine and Rhine, the Norse Vikings could raid, trade, and settle inland across Europe (Mazet-Harhoff, 2010) (Mazet-Harhoff, 2010:95 Figure 2). Subsequent to this activity, Norse settlements were established in Scotland, Normandy (Renaud, 2008), and Brittany (Price, 2008) during the 10^th^ century.

There were also Viking incursions into the Iberian peninsula and around the Mediterranean which began in the 840s CE (Christys, 2015). There are few sources that describe later Viking activities in southern Europe; however, Christys’s (Christys, 2015) timeline shows that there were mid-to-late 10th-century Viking attacks in Spain and Portugal.

There were also north and eastbound activities towards, respectively, the North Calotte (the name given today to the transnational area of northernmost Scandinavia) and the White Sea (Bjarmeland). Already by 890 CE, travel to Bjarmeland has been documented (Bratrein, 2018). It has been confirmed that the extensive trade of goods originating in this region increased during the following centuries (Storli, 2018). Although Hadsel in Vesterålen, northern Norway, was raided during the mid-10^th^ century (Bratrein, 2018), there is evidence that points to activity in the north being geared towards economic resource extraction, thus differing from activity in the Insular area and continental Europe. However, more research on this topic is needed to clarify such assumptions. There are few contemporary written sources that reveal anything about migration into Scandinavia during this period. Even so, aDNA research (Margaryan et al., 2020:393 (Margaryan et al., 2020)) and archaeological material studies (Glørstad, 2012) collectively support such mobility, as western regions of Scandinavia did indeed receive ancestry from the British Isles during this period.

Except for the written sources reporting Viking attacks on Portland (789) and Nantes, France (843) in which the origin of the Vikings is specified as being, respectively, the Hordaland and Vestfold regions in Norway, few sources specifically identify the region of origin of Vikings (Christys, 2015). These few written sources reveal next to nothing about the extent of mobility throughout Viking Age Scandinavia, or about whether a more fine-tuned regional variability prevailed within Scandinavia. Moreover, we currently know very little about the social structure and organization of the groups migrating out of Scandinavia or those who were mobile within this area.

Viking Age Scandinavian burial customs show clear regional and chronological variation. In Norway, inhumation is more prevalent in northern regions, while cremation is more common in the south (Sjøvold, 1962, 1974; Stylegar, 2009). The total number of both inhumations and cremation burials covering this time span is currently not available; however, the number of inhumations known from the Norwegian Schreiner Collections biobank (The Schreiner Collection database, 2022a) is much smaller than the number of known cremations, leaving a limited record suitable for bioarchaeological studies. Considering these factors alongside skeletal taphonomy, care has been taken in this study to ensure representativity with regard to biological sex, age at death, and geography.

**Figure 1:**
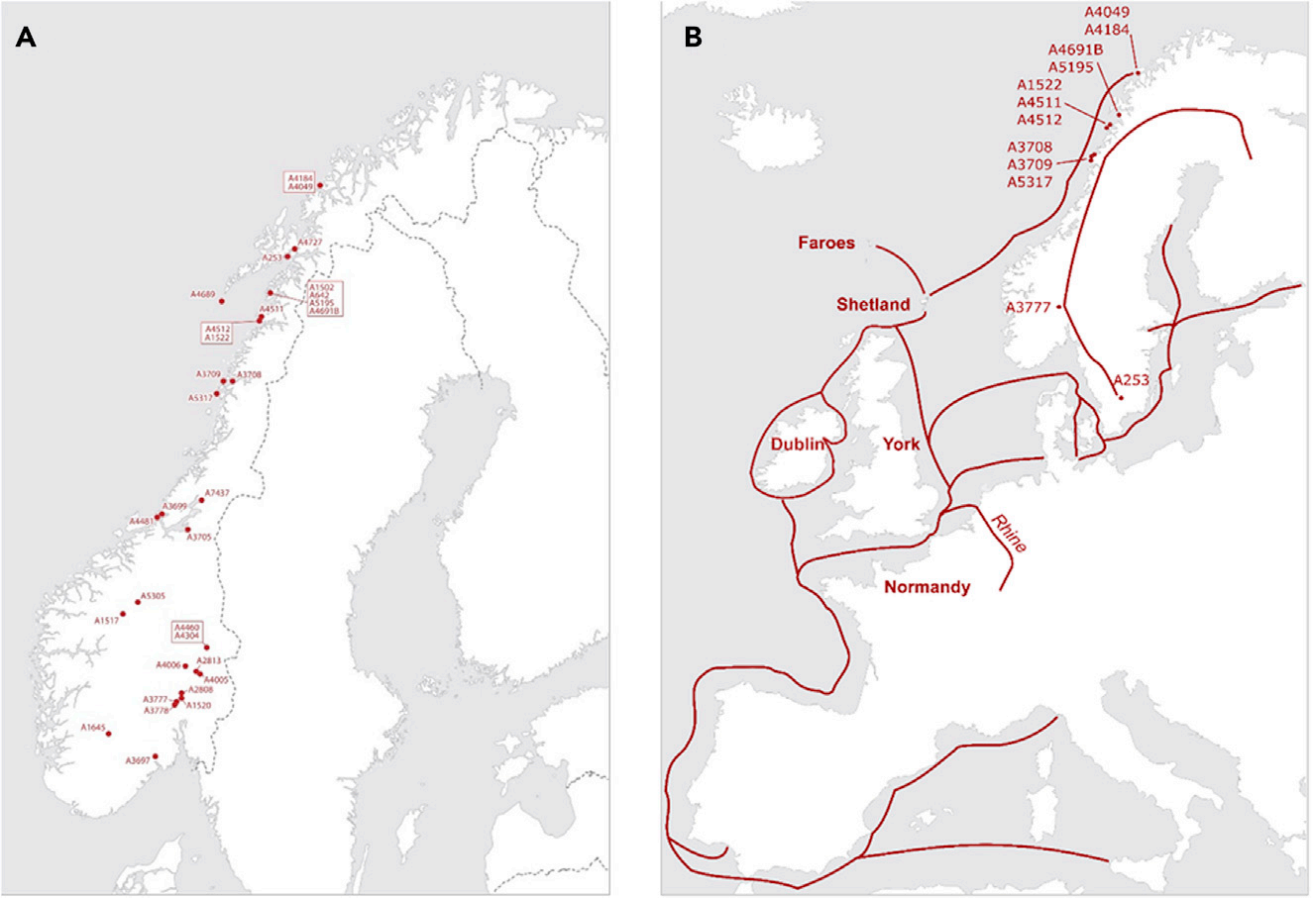
Grave contexts and mobility patterns (A) Geographical distribution of the inhumation graves in this study. (B) An overview of common Viking Age routes including the geographical areas of origin identified through δ^18^O results.

In order to explore the character and mobility of the Late Iron and Viking Age individuals buried in Norway, we isotopically analyzed the remains from 30 of the inhumation graves. All samples had previously been subjected to ancient DNA analysis as part of a larger international dataset (see Margaryan et al., 2020 Figure 1A:391) (Margaryan et al., 2020). We analyzed enamel carbonate (*δ*^18^O, *δ*^13^C_carb_) and collagen (*δ* ^13^C, *δ* ^15^N) from 30 individuals deriving from Late Iron Age and Viking Age graves distributed widely throughout Norway (Figure 1A). Oxygen isotopes in human hard tissues are an established marker for studying residential changes across the life course. Oxygen isotope values (δ^18^O) in bones and teeth are related to climate and local water sources (Chenery et al., 2014; Pellegrini et al., 2016). Meanwhile, δ^13^C and δ^15^N in collagen reflect the dietary composition and primary protein source at the time of tissue formation/remodeling (see STAR methods for greater detail). Given that these isotopic values present a finer scale of sub-structuring than genomics, this analysis makes a substantial contribution to our understanding of individuals’ mobility and life history. Analysis of the combined aDNA and isotope datasets facilitates the study of both mobility and diet composition between age groups and gender, and thus permits a closer investigation of the potential composition of the migrating groups, the degree of residency versus mobility, and the individual’s living conditions.

Archaeological research has tended to construct rigid social roles for Viking Age people based on interpretations of the archaeological material (Løkka, 2014). For instance, keys found in burial contexts are both concrete and symbolic examples, as the interpretation of keys has tended to identify the interred individual’s sex as female and social role as a housewife (Pantmann, 2014). Conversely, the presence of weapons in graves is often assumed to correlate strongly with maleness, thus amplifying the dichotomous tendencies of cultural historical research, and reinforcing associations of hegemonic masculinity with militarism (Raffield, 2019). These examples reflect preconceptions of the stationary woman and the mobile male during the Viking Age (Krzewińska et al., 2015). As mobility is a core parameter for defining the Viking era, the representation of sex/gender roles within this framework, especially in relation to the agency of mobility, is a central consideration.

Biomolecular research in archaeology has tended to see variations in mobility and diet as being based on gender differences. Naumann’s (Naumann, 2014) multi-isotope research sampled human remains from the Viking Age to investigate diet (δ^13^C/δ^15^N) and mobility (^87^Sr/^86^Sr). This research indicated slight dietary differences between women and men, the latter considered to have had a higher degree of marine-based food intake. This disparity has been interpreted both as evidence of possible gendered labor division and as reflecting mobility (Naumann, 2014; Naumann et al., 2014a). Therefore Naumann’s (Naumann, 2014) research concluded that men had a higher degree of mobility than women during the period.

Recent biomolecular studies on human remains have nuanced the gendered view of Viking Age social organization and given greater insight into the complexity of this period’s socio-cultural organization. The now famous case from Birka, Sweden (Hedenstierna-Jonson et al., 2017), which identified a female Viking warrior, and the weapon grave in Suontaka Vesitorninmäki, Finland, where an individual likely with Klinefelter’s syndrome was buried with both weapons and jewelry (Moilanen et al., 2021), are both examples of cultural diversity in Viking Age societies. The present study aims to further investigate correlations between mobility, diet, and biological sex in Norway during this period, and to evaluate whether or not there are any regional patterns latent in the multi-isotope (δ^13^C_carb_, δ^13^C/δ^15^N_coll_, δ^18^O) evidence. Following this, we hope to assess to what extent the analysis supports a deeper understanding of social structure and organization in Norway at this time.

## Results

To address Viking Age mobility and diet variation, we performed a multi-isotope analysis on 30 human individuals that had previously been characterized genetically (Margaryan et al., 2020). The majority of the skeletons (n = 27) were confirmed through radiocarbon dating and grave goods typologies to be from the Late Iron and Viking Age; three individuals were found to be incompatible with the period (Table S3 in supplementary). Individual A4689 (Table S3 in supplementary) is nudged into the medieval period based on the marine reservoir correction; however, their grave goods are consistent with a Viking Age date (Figure S1 in supplementary). As seen in Table S3 (in supplementary) there are outliers dating to the Norwegian Early Iron Age (500 BC–550 CE), the Bronze Age (1700–500 BC), and the modern era (after 1500 CE). The radiocarbon results were obtained after isotopic analyses were complete, therefore all the data are reported here, but individuals with outlying data were not interpreted as part of our Viking Age analyses later in discussion. Terrestrial and mixed marine columns indicate dates derived from ^14^C measurements, whereas the archaeological dating originates from archaeological reports.

### Interpretation of diet results

To investigate dietary variation in our material, we analyzed δ^13^C and δ^15^N in human collagen (see STAR methods for details). More negative δ^13^C values are suggestive of a terrestrial C_3_-based diet, whereas less negative values can indicate increased marine resource consumption (Chisholm et al., 1982; Tauber, 1981). As δ^15^N increases up the food chain, it is a useful proxy for increasing protein consumption, and when used in conjunction with δ^13^C it can therefore indicate the source of that protein (terrestrial vs marine) (Hedges and Reynard, 2007). However, manuring and varied watering regimes for plants can lead to ^15^N enrichment which can be passed up the food chain (Bogaard et al., 2007; Lightfoot et al., 2020). Nutritional/physiological stress such as famine, pregnancy, and breastfeeding can also elevate δ^15^N values in human tissues, so caution is needed when interpreting results (Fuller et al., 2005, 2006).

**Figure 2:**
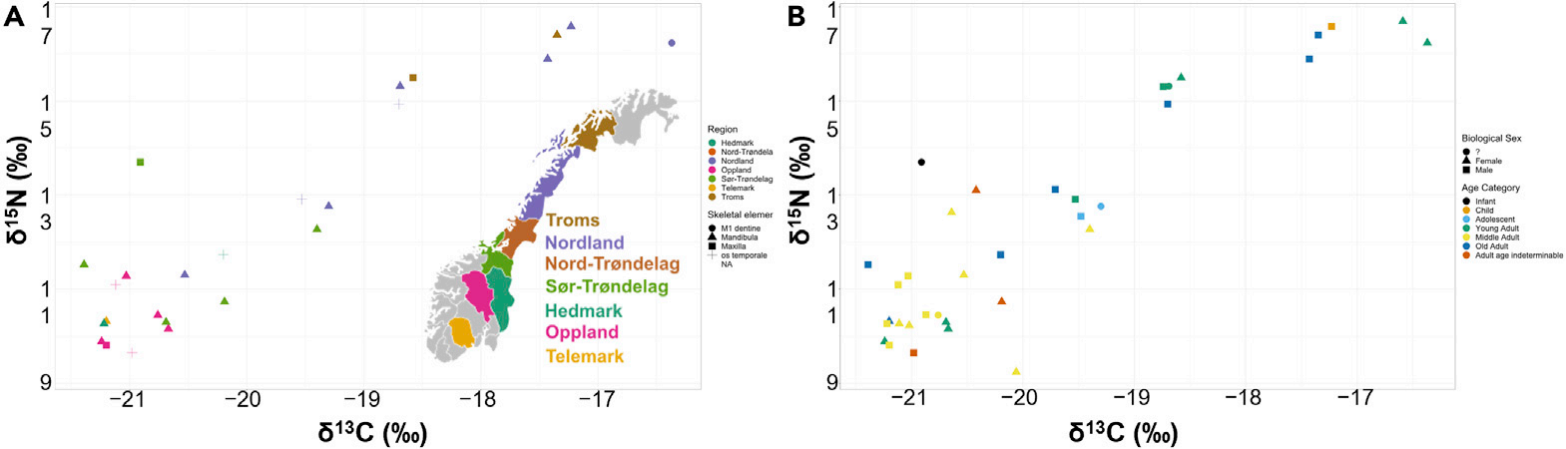
Stable Isotopic diet patterns (A) Scatter plot of bone collagen δ^13^C and δ^15^N values, colored by region, with shapes indicating skeletal element. Figure 2B: Scatterplot of bone collagen δ^13^C and δ^15^N values, colored by age with shapes indicating biological (Margaryan et al., 2020) and osteological sex.

Collagen δ^13^C values in our samples range from −21.39 to −16.37‰ (mean −19.72 ± 1.47‰) and δ^15^N collagen values from 9.24 to 16.69‰ (mean 12.66 ± 2.46‰), as seen in Figure 2A. These large ranges in collagen δ^13^C and δ^15^N values (4.65 and 7.45‰, respectively), accompanied by large standard deviations, suggest that these individuals were exploiting vastly different food resources and ecosystems across several different trophic levels. Some of this variability can be explained regionally (Figure 2A), with data from burials in the northern regions of Nordland and Troms being on average consistent with regular marine protein consumption. However, when these individuals are excluded, the δ^15^N collagen values have a range of 4.46‰, which is within the expected range of trophic-level enrichment (3–5‰), so likely represent small differences in proportional amounts of protein consumed, but nothing on a large scale (Bocherens and Drucker, 2003; Hedges and Reynard, 2007).

Some of this variation can be further explained by the skeletal element sampled and the age of the individuals. Whilst there is no clear pattern of isotopic offsets between skeletal elements, the sole first molar analyzed shows a high degree of isotopic enrichment for both δ^13^C and δ^15^N. As the first molar forms during infancy (Table S2 in supplementary), this could be owing to a breastfeeding signature (i.e. fractionation in human breast milk and trophic enrichment between the child and mother) as well as other physiological differences in infants, complicated isotopic routing in breast milk and the gut microbiome (Beaumont, 2020; Beaumont et al., 2015, 2018; Burt, 2013; Fuller et al., 2006; Haydock et al., 2013; Kendall et al., 2021; Reynard and Tuross, 2015).

**Figure 3:**
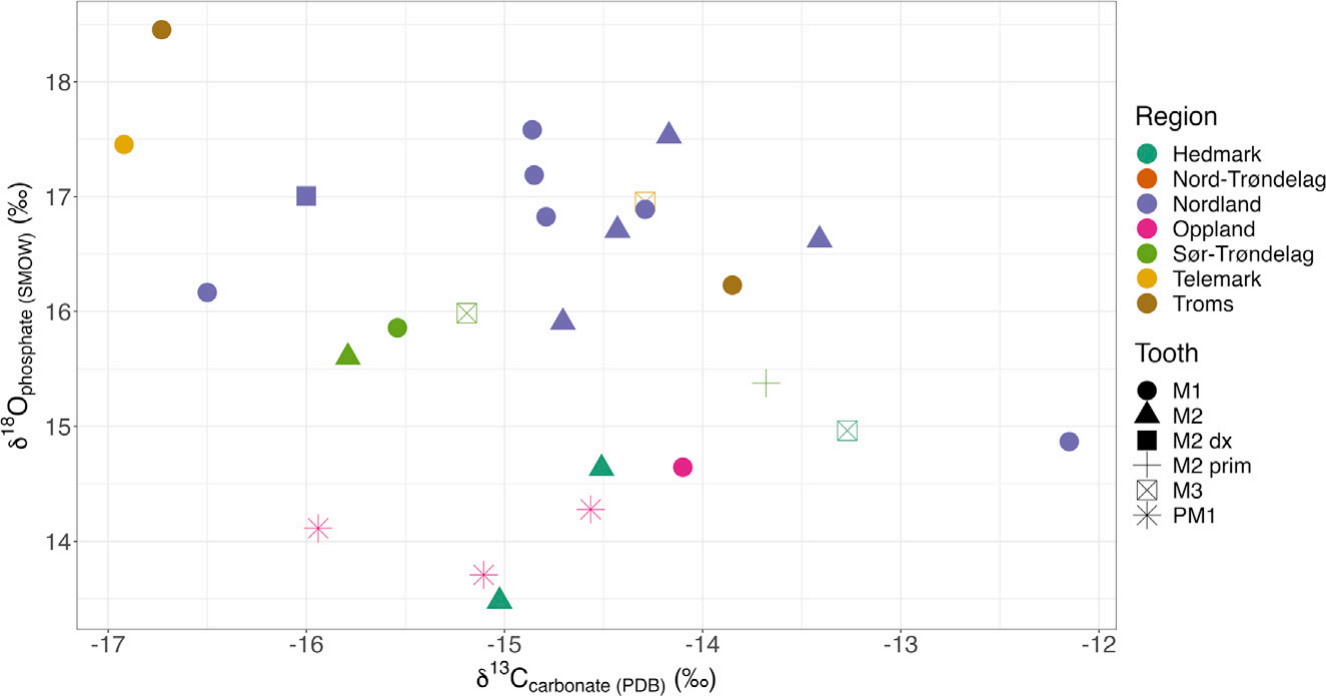
Scatterplot of enamel δ^13^C_carbonate_ and δ^18^O_phosphate (SMOW)_ values, colored by region with shapes indicating tooth sampled

Figure 2B demonstrates that there is some relationship between the age at death of the individual and isotopic enrichment, but this is hard to disentangle from the regional variation with the sample sizes we are dealing with. Nor could we observe a definitive relationship between biological sex and diet (Figure 3B).

The δ^13^C_carb_ values from tooth enamel in the sample range from −16.92‰ to −12.15‰ (mean −14.79 ± 1.11‰) which mirrors the broad range in collagen δ^13^C values described above (see Figure 3). This suggests that whole diets in these individuals range from reliance on primarily terrestrial and/or freshwater resources to diets heavily reliant on marine resources (Ambrose and Norr, 1993; Clementz et al., 2009; Kusaka, 2019; Kusaka et al., 2015).

Overall these results demonstrate regional dietary differences in Viking Age Norway, with northern regions being more heavily reliant on marine resources with no clear dietary differences between the sexes or age groups.

### Regional differences in mobility revealed through δ^18^O values

In order to investigate if the individuals in our study showed evidence of mobility during their lifetimes, we analyzed tooth enamel carbonate. The δ^13^C_carb_ enamel results were described briefly above in relation to diet, but are also used here to contextualize and illustrate δ^18^O variability, as very few individuals have ^87^Sr/^86^Sr data from previous studies, and because further strontium analysis was beyond the financial scope of this project (Naumann et al., 2014b, 2019; Price and Naumann, 2014). δ^18^O was analyzed from the carbonate portion of the tooth enamel as described in the STAR methods; the δ^18^O_carb_ values of individuals here range from −8.21‰ to −3.54‰ (mean −5.88 ± 1.24‰). When converted to δ^18^O_phosphate_ via the Chenery et al. (2012) (Chenery et al., 2012) equation as per Leggett et al. (2021) (Leggett et al., 2021) the range is between 13.48 and 18.45‰ (mean 15.96 ± 1.32‰, see Figure 2), and when converted to drinking water values, also using Chenery et al. (2012) (Chenery et al., 2012), the range is −12.95‰ to −5.29‰ (mean −9.13 ± 2.03‰). These ranges and standard deviations are far larger than would be expected if the individuals were local to their regions of burial, and are not fully compatible with the current oxygen isoscapes for Norway (Bowen and Revenaugh, 2003; Lightfoot and O’Connell, 2016). To counteract possible regional biases and give a better understanding of migration in relation to the place of burial, Δ^18^O_dw-MAP_ values are therefore also used as per Leggett et al. (2021) and Leggett (2021a, 2021b) (Leggett, 2021a; Leggett et al., 2021).

**Figure 4:**
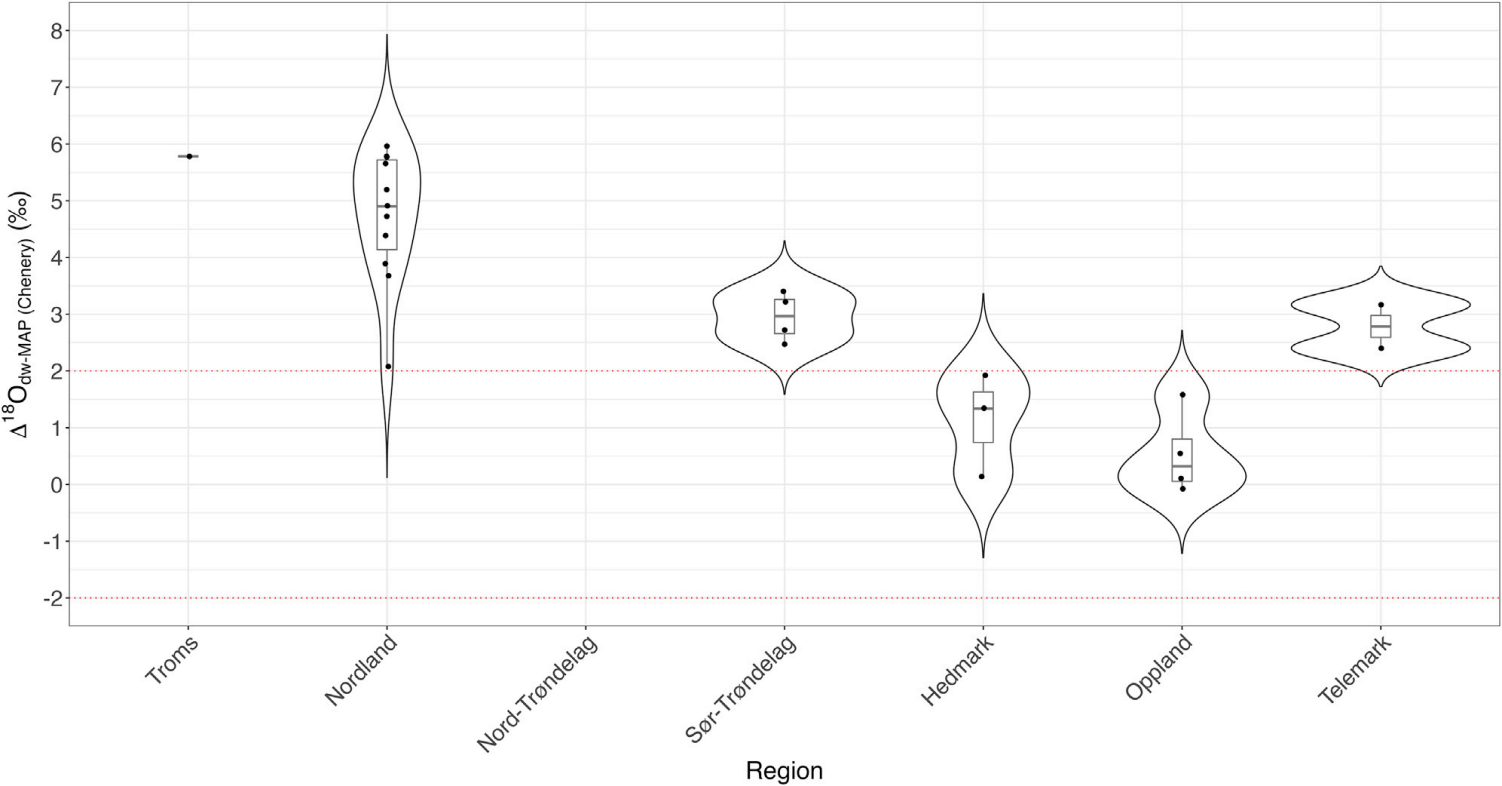
Violin plot of enamel Δ^18^O_dw-MAP_ values by region with red dotted lines indicating the ±2‰ range for being “local” to the grave site The boxes and whiskers indicate interquartile range and 1.5x interquartile range, respectively

**Figure 5:**
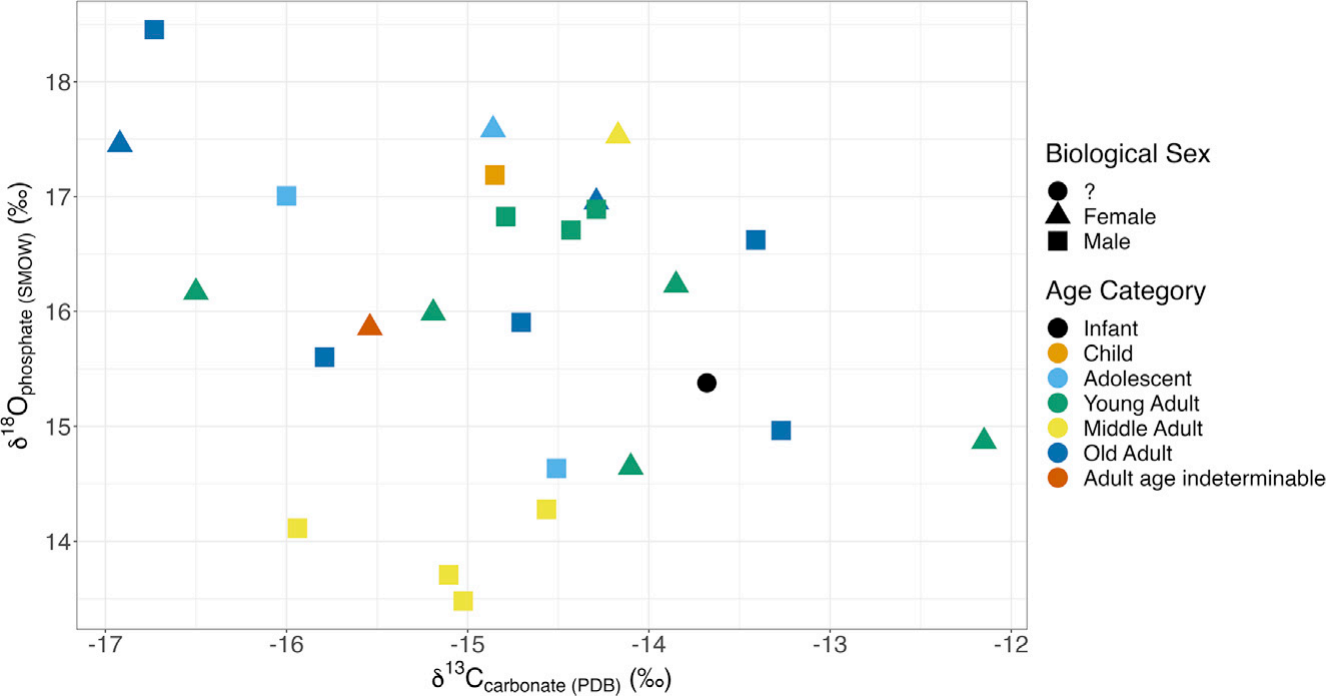
Scatterplot of enamel δ^13^C_carb_ and δ^18^O_phosphate (SMOW)_ values, colored by skeletal age with shapes indicating biological sex; “?” indicates an unsexed individual

Figure 3 highlights the regional biases in the sample and tooth types analyzed. Individuals buried in Nordland are quantitatively dominant; however, there do seem to be regional differences in δ^18^O_phosphate_ values, with Oppland and Hedmark individuals having lower values than those in other counties. Tooth selection could be playing a role in the isotopic variation with different teeth representing different crown formation timings (e.g. M1 crowns forming from birth to age three versus M3s forming from approximate age seven to sixteen) (Table S2 in supplementary) (Scheid, 2007). Most samples are M1s, and these tend to have higher δ^18^O_phosphate_ values than the other teeth and equate to larger and more positive Δ^18^O_dw-MAP_ values in Figure 4. This could be “brewing and stewing” and further isotopic fractionation at play from breastfeeding (Brettell et al., 2012; Kendall et al., 2021; Pederzani and Britton, 2019); however, the fact that other teeth from the same regions tend to plot alongside the M1s suggests that this fractionation may be minimal and that the M1s are displaying true migration signatures. This is further supported by Figure 5 where no clear differences between age groups can be seen.

Figures 3 and 4 therefore suggest that there may be regional differences in the degree of migration. Here, Nordland and Troms display significantly higher values than the rest of Norway. Individuals buried in Hedmark and Oppland fall entirely within the local range for their burial places, whereas burials in all other regions fall above +2‰ Δ^18^O_dw-MAP,_ which means we must weigh up the evidence for migration versus “brewing and stewing” as the cause for this positive fractionation. As mentioned above, this could be evidence of regional differences in the consumption of “brewed and stewed” foods and drink, and/or of breastfeeding signatures in early forming teeth, and/or of signatures of individuals moving into these regions from isotopically enriched regions which were possibly warmer than these northern regions of Norway. Interestingly, unlike in other parts of western Europe, there is no evidence here, based on oxygen data alone, of migrants from the more northerly regions migrating further south in Norway—if anything the opposite appears to be the case here (Leggett, 2021a). However, when combined with the limited existing ^87^Sr/^86^Sr data from Naumann’s work in double-isotope provenancing models (Figure 7), possible regions of origin are surprisingly varied and do include northern regions of Fennoscandia (Bataille et al., 2021; Colleter et al., 2021; Naumann, 2014; Price and Naumann, 2014).

## Discussion

We highlight here regional variation in both diet (δ^13^C/δ^15^N) and mobility (δ^18^O) in Viking Age Norway. The individuals from northern regions demonstrate a higher degree of marine protein consumption, a phenomenon that declines on a southerly gradient, indicating regional dietary practices and resource availability. Additionally, in contrast to Naumann’s (Naumann, 2014) work, we do not find in our material any dietary differences between the sexes in this study (Figure 2B). Of the 24 δ^18^O samples, 70.83% (N = 17) display either inter-regional or long-distance mobility in relation to their place of burial. Seven individuals display isotopic values similar to their place of burial. The long-distance mobility patterns are particularly evident in coastal regions of northern Norway (Figure 1A), which suggests regional migration practices throughout the Viking Age.

Overall, the political structure of Norway prior to statehood may have been one of the causes of interregional and long-distance trade and incursions demonstrated in the present evidence. During this period Norway was ruled by chieftains who had control over large areas and plentiful natural resources. This social system was to a large degree maintained through reciprocity between the chieftain and their subjects that sustained loyalty during conflicts in Norway and abroad (Berglund, 1996; Bratrein, 2018). One example of such reciprocity may be the silver neck ring found on the Island of Senja in northern Norway. Its inscription is quite telling: “*We went to Frisia and fought the warriors, and we shared the booty between us”* (Stylegar, 2015). Still, there were probably several types of alliances of war bands or traders which were not necessarily organized by a chieftain; the highly multicultural 9^th^-century Viking “Great Army” in England is one such example (Hadley and Richards, 2016; Jarman et al., 2018; Raffield, 2016). Therefore, it is not surprising that we have identified both highly localized dietary patterns and multi-scalar mobility in the isotopic data.

Our multi-isotopic dataset corresponds directly to the picture obtained from the majority of genetically researched Norwegian individuals, designating a “Norwegian-like” movement primarily towards the North Atlantic region (Margaryan et al., 2020, Figure 4:394) (Margaryan et al., 2020). As seen in Figure 1A, the human remains analyzed here represent a considerable swathe of Norway, which, through multi-isotope data (δ^18^O, δ^13^C, δ^15^N, ^14^C), allows us to discern both regional and cultural practices in relation to diet and mobility.

Radiocarbon dating (Figure S1 and Table S3) shows that there was mobility throughout the early periods, defined by small-scale raids, as well as the mid-to-late Viking Age characterized by overwintering encampments and overseas settlement. The oxygen isotope patterns that have been revealed include areas known for having a Viking presence, encompassing southern Scandinavia, and most of temperate Europe (Figure 1B). Similar patterns are clear in Leggett’s research (Leggett, 2021a) on early medieval individuals in England, where multi-isotope results disclose reciprocal migration between the Mediterranean region and England. As well as highlighting far-reaching mobility, the present study also reveals regional movements within Norway. A pioneering oxygen isotope study (δ^18^O) on the medieval (1050–1300 CE) population in Trondheim, a very important town in Viking Norway, has also shown an influx of children and reveals both regional and long-distance mobility into this urban area following the Viking Age (Hamre and Daux, 2016).

**Figure 6:**
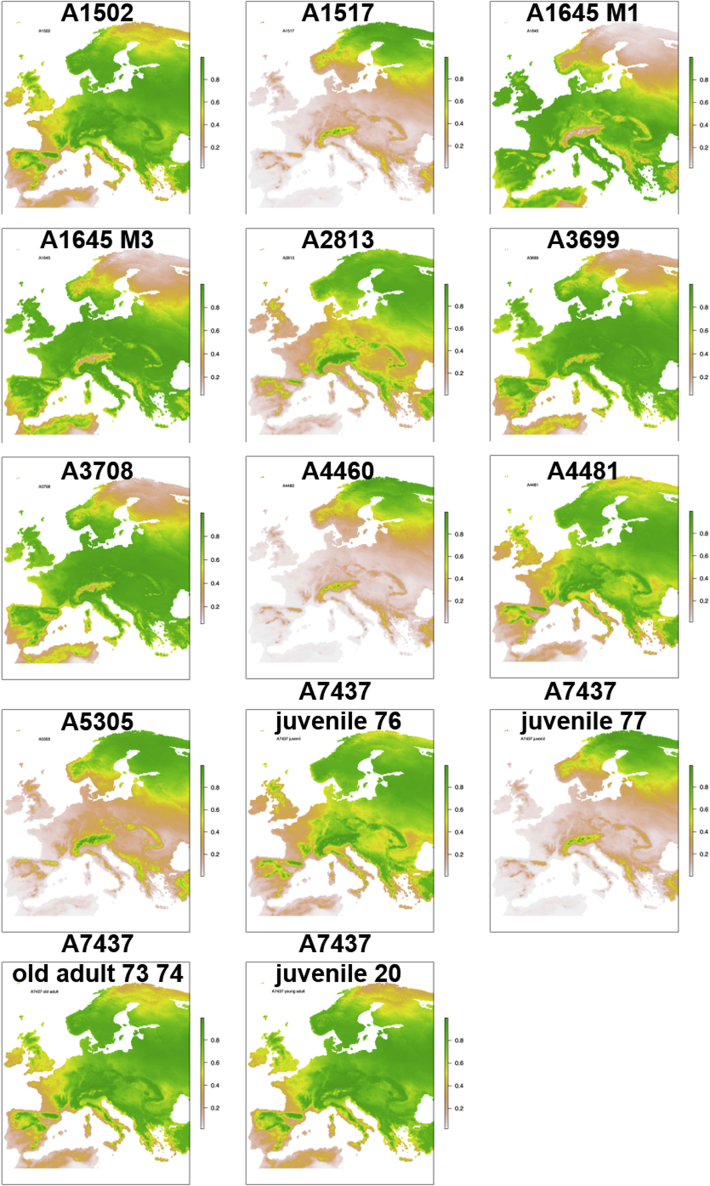
Single isotope (δ^18^O) probabilistic origin maps for individuals in this study

**Figure 7:**
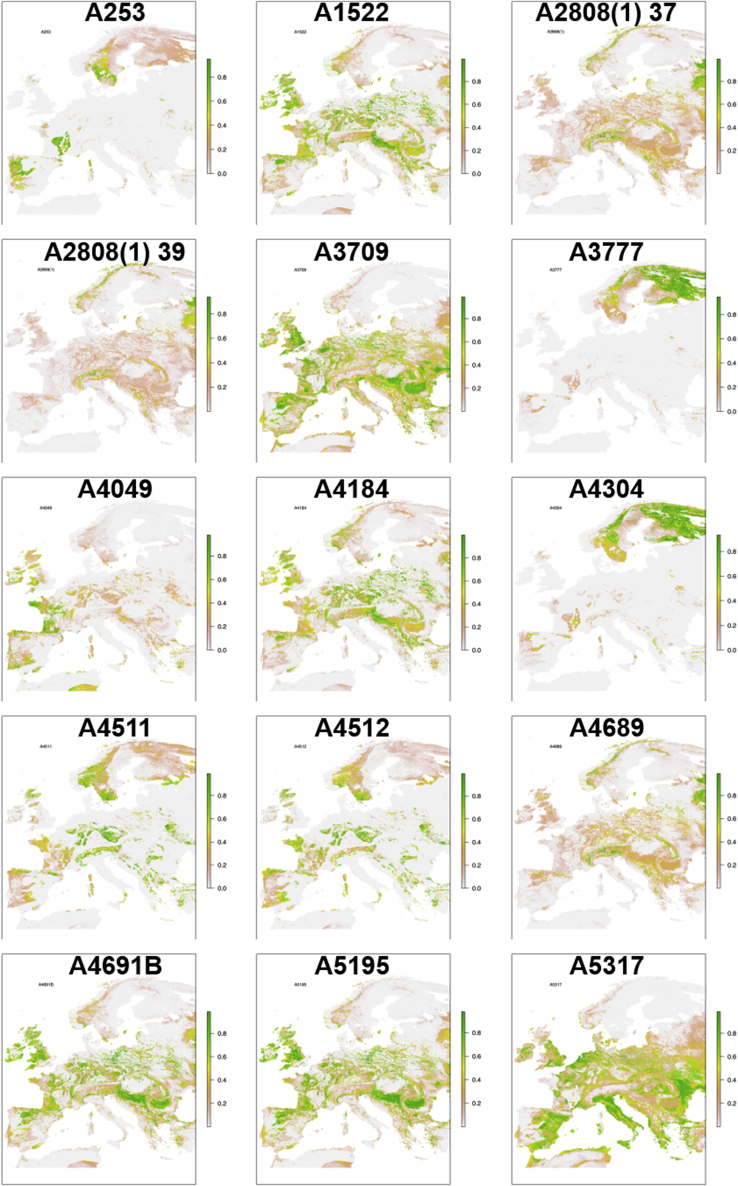
Double isotope (δ^18^O-^87^Sr/^86^Sr) probabilistic origin maps for individuals in this study Strontium data is from Naumann and Price and Naumann (2014) (Naumann, 2014; Price and Naumann, 2014), oxygen data, except from individual A253 which oxygen data is from Price and Naumann (2014) (Price and Naumann, 2014), the rest is from this study (see Table S4 in supplementary).

For some of the individuals, we have additional strontium data from previous work (Naumann, 2014; Price and Naumann, 2014) which in addition to δ^18^O gives geological provenancing information for individual mobility (Figures 6 and 7). The application of single (δ^18^O) and double-isotope (δ^18^O and ^87^Sr/^86^Sr) predictive origin models in R (Bataille et al., 2021; Colleter et al., 2021; R Development Core Team, 2021) was used to generate illustrative maps of potential regions of childhood origins for the individuals analyzed here and add greater interpretative power to the isotopic data. For a full explanation of the models, see Bataille et al., (2021) (Bataille et al., 2021). Through the application of these models, it is evident that, with the exception of A4049 indicating mobility towards warmer areas such as the Bay of Biscay, coastal Spain, and the Mediterranean, there was an emphasis on temperate Europe. Specifically, Ireland, Britain, France, Flanders, the Netherlands, and southern Scandinavia appear to have been likely geographical areas of residence (for both sexes) during the migrants’ early lives (Figure 1B).

There are fewer examples of mobility towards the Northern Calotte in this study. Even so, for example, isotope values (δ^18^O, ^87^Sr/^86^Sr) (Figure 7, Table S6 in supplementary (Price and Naumann, 2014)) for A253 buried in Ytterstad, Nordland indicate that at some point during childhood this person spent time in areas in southern Sweden or possibly southern Norway. This individual also exhibits *variola* virus strain which may have an affinity to a contemporary individual buried in Öland in southern Sweden (Mühlemann et al., 2020). Pointing towards a north-south medium range mobility, A3777 (C24243), buried in the southern part of Norway, had been in the Northern Calotte area sometime during childhood.

Mobility patterns unveiled by the present study are supported by the archaeological material found in some of the burials discussed in this study. An example of such is the sword type L of Insular origin (Androshchuk, 2014) identified in the burial of A5305 (C35586). For both A4511 and 4512 (both Ts4306), there is some overlap between the prevalence of the sword types V and Y, originating in southern Scandinavia and northern Europe, respectively (Androshchuk, 2014). These individuals’ childhood mobility further highlights links across Northern Europe and the Atlantic archipelago. An interesting exception is A4049 (Ts3525) who was buried with a common sword-type from the North Sea zone; however, isotopic evidence suggests he spent his childhood in southern Europe and the Mediterranean (for an overview of swords and distribution pattern, see Table S5 in supplementary). Even though we cannot entirely rule out Atlantic Fringe zones as the place of origin for this person, the correlation between the multi-isotope results and this individual’s radiocarbon date lines up with Viking activity in southern Europe and further supports our interpretation (Figures 6 and 7, Tables S3 and S4 in supplementary) (Androshchuk, 2014; Christys, 2015; Petersen, 1919).

Both biomolecular and historical sources show extensive geographical mobility of both family groups and war bands in the first millennium AD (Cooijmans, 2020); trade between southern Scandinavia and Francia, with possible trade corridors into the Mediterranean, is indicated by Cooijmans (2020). Even though the Frisians are represented as principal actors, radiocarbon dating together with δ^18^O data indicate early Viking Age contact between Hålogaland in northern Norway and continental Europe during this period (Figures 6 and 7 and Tables S3 and S4 in supplementary).

It is abundantly clear, with this new isotopic data integrated with earlier evidence, that people were highly mobile during this period. A4184 and A4049, which both demonstrate δ^18^O values consistent with mobility since childhood (Table S4 in supplementary), were buried near Hillesøy-Kvaløya in Troms county. The presence of a trading center in this area is supported by the foreign character of the area’s Viking Age archaeological material (Mikkelsen, 2019). Another find from this region that indicates long-distance contact is the Rønvik hoard, deposited, sometime after July 949 CE, close to burials A4511 and A4512, both of which show far-reaching mobility (Table S4) (Spangen, 2005). This hoard included Late Anglo-Saxon and Arabic Kufic coins, and a silver neck ring. The recognition of Sandtorg as a northern market and trading center is important for the understanding of the mobility identified in Hålogaland during the Viking Age. This all further supports the hypothesis that there was frequent mobility of people, goods, and ideas into and out of Norway during the Viking Age, with wide-ranging connections across Europe and further afield.

The multi-isotopic results have demonstrated a high degree of regional patterning for both mobility and diet in Norway. Furthermore, we have shown that there were very few isotopic differences between the sexes to support gendered diet or migration. This has interesting implications for our understanding of mobile family structures and wider social organization during this period.

As human teeth develop over several years (Table S2), one has to be careful, when using isotope studies, not to equate these chemical signatures with definitive movement at a certain age or with definitive origins. However, the isotopic values indicate that, for a period of time, a number of the analyzed “Norwegian-like” Viking individuals spent part of their childhood at a great distance from their place of burial in Norway. Another recent strontium (^87^Sr/^86^Sr) study has also revealed similar patterns of medium and far-reaching mobility by children along confirmed Viking routes (Naumann, 2014). The material included a young boy (A253/C18558) who was buried in Lødingen, in northern Norway, who, according to his ^87^Sr/^86^Sr values, had spent some time in Southern Norway (Naumann, 2014). As mentioned earlier in this study the double isotope (δ^18^O-^87^Sr/^86^Sr) results (Figure 7) indicate, however, that parts of southern Sweden are also likely areas in which A253 could have spent some time during the early Viking Age. Migration and mobility among young people are discussed in Hadley (Hadley and Hemer, 2011), where Norse children, through the Anglo-Saxon Chronicle, are identified within the context of Viking armies, giving them important roles as social agents.

As some of the individuals in this study were quite young when the isotopic signatures were deposited in their enamel during their journeys (Table S4), we assume that they traveled as members of a family group. And if this is the case, the radiocarbon dating indicates that family group mobility was prevalent in Norway throughout the Viking Age.

### Conclusions

This study offers insights into both macro-scale socio-cultural paradigms and regional-level patterns. The picture we get of Norwegian Viking Age society is far more detailed than anything we can obtain from documentary sources and material culture studies. Even though we cannot equate childhood origin and childhood movement simplistically from the isotopic data, the difference in climatic conditions indicated by isotopic values when they run counter to expected values if, the individual concerned always lived in the region where he or she was buried, suggests that some of the individuals may have been part of mobile family groups (Krzewińska et al., 2015; Løkka, 2014). Similar results have also been demonstrated for the Viking Great Army in Britain by Hadley (Hadley and Hemer, 2011). Moreover, our data strongly suggest that mobility, one key facet defining the Norwegian Viking Age, was not a male prerogative, and this is supported by research showing a Scandinavian maternal lineage (mtDNA) influx into Orkney and Shetland during the Viking Age (Krzewińska et al., 2015). Both examples co-contemporize women, children, and men as mobile during this period.

Another significant finding is that the observed dietary variation reflects regional resource exploitation rather than diet differences between the sexes. Ongoing biomolecular and archaeological research also supports geographical differences in the exploitation of ecosystems during this period. The populations of northern and Arctic Norway had a heavy reliance on fish and marine mammal food intake (Barrett, 2016). Such exploitation patterns are clear by the 12^th^ century as part of the *Fish Event Horizon*, seen in the Viking diaspora that was likely owing to the establishment of large-scale fishing industries in Norway (Barrett, 2016). However, even though written sources do not mention cod trade prior to the early 12^th^ century, archaeological research does indicate the prior organization of cod fishing in Scandinavia (Nielssen et al., 2016). Marine fisheries are central to understanding some of the regional diets identified from both this article and Leggett’s research (Leggett, 2021a, 2021b).

This interpretation of mobility is supported not only by the archaeological materials’ international character prior to, and during, the Viking Age but also by genetics (Krzewińska et al., 2015; Margaryan et al., 2020) and by the isotopic results presented and discussed in the article. Genetic results also reveal higher levels of genetic heterogeneity in coastal regions during this period.

As the δ^18^O analysis, together with written sources (Cooijmans, 2020), shows, Norse family groups were widespread across areas of a known contact. Even though the isotopic data do not shed light on specific activities that were undertaken by the family groups, such as trade and raids, for instance in the Insular World and in continental Europe, this study underlines that such enterprises likely included women, children, and men (see Cooijmans, 2020 for an extensive overview) (Cooijmans, 2020). For instance, Stalsberg’s (Stalsberg, 1991) research on weighing equipment found in female Scandinavian-like burials in Russia, Sweden, and Norway highlights the critical role which Viking Age tradeswomen played. This mobility may have also included women partaking in raids and battles; several sources mention female Viking warriors. For instance, they are mentioned in one battle between the Rus and the Byzantines in 970 CE (for a substantial overview, see Moen (2010). Another example from the Norse Hervara saga is Hervǫr, who also went into battle (see the extensive overview in Gardeła (2021).

In line with previous work (Hedenstierna-Jonson et al., 2017; Moilanen et al., 2021), this study complements other research with its interdisciplinary approach. We have combined critical bioarchaeological and archaeological interpretations to re-assess gender roles, diet, and mobility in Viking Age Norway. Here we bring to light several socio-cultural and regional phenomena distributed over the spectrum of the Viking Age, the possible presence of children in overseas activities—implying that both girls and boys were important within the family structures—and the fact that women shared economic and military roles with men, demonstrated via mobility patterns and through similar dietary signatures. To conclude, the results from both diet and mobility analyses indicate that a fruitful direction for further Norwegian Viking Age research lies within the aspect of regionality.

### Limitations of the study

The main limitation of the study is the limited number of individuals available for sampling.

First, graves from the region and period are normally in the form of cremation burials, and our study is limited to inhumation burials. Second, owing to taphonomy and legal restriction, some areas of Norway, such as the western part of Norway are not represented in this study, and our findings may therefore be limited geographically. This is the western part of Norway, the counties of Møre og Romsdal, Vestland, and Rogaland, as well as Finnmark county in northern Norway. Thus, there is a possibility that our findings may not be applicable to other geographical regions, or to individuals who were cremated rather than inhumed. These caveats may affect the trajectories implied in the discussion on the basis of the results of the study.

## STAR★Methods

### Key resources table

**Table:** 

REAGENT or RESOURCE	SOURCE	IDENTIFIER
**Biological samples**		

Ancient skeletal remains	This Study	A253/C18558
Ancient skeletal remains	This Study	A642/T5105
Ancient skeletal remains	This Study	A1502/C18035
Ancient skeletal remains	This Study	A1517/C14690
Ancient skeletal remains	This Study	A1520/C21852
Ancient skeletal remains	This Study	A1522/C14554
Ancient skeletal remains	This Study	A1645/C21794
Ancient skeletal remains	This Study	A2808(1)/C4438
Ancient skeletal remains	This Study	A2808(2)/C4438
Ancient skeletal remains	This Study	A2813/C17564
Ancient skeletal remains	This Study	A3697/C23941
Ancient skeletal remains	This Study	A3699/T13363
Ancient skeletal remains	This Study	A3705/T2327
Ancient skeletal remains	This Study	A3708/T9366
Ancient skeletal remains	This Study	A3709/T12578
Ancient skeletal remains	This Study	A3777/C24243
Ancient skeletal remains	This study	A3778/C24297
Ancient skeletal remains	This Study	A4005/C25552
Ancient skeletal remains	This Study	A4006/C25720
Ancient skeletal remains	This Study	A4049/Ts3525
Ancient skeletal remains	This Study	A4184/Ts3639
Ancient skeletal remains	This Study	A4304/C26737
Ancient skeletal remains	This Study	A4460/C27338
Ancient skeletal remains	This Study	A4481/T16298
Ancient skeletal remains	This Study	A4511/Ts4306
Ancient skeletal remains	This Study	A4512/Ts4306
Ancient skeletal remains	This Study	A4689/Ts5252
Ancient skeletal remains	This Study	A4691b/Ts5287
Ancient skeletal remains	This Study	A4727/Ts5656
Ancient skeletal remains	This Study	A5195/Ts7659
Ancient skeletal remains	This Study	A5305/C35586
Ancient skeletal remains	This Study	A5317/T20545
Ancient skeletal remains	This Study	A7437/T20248
Ancient skeletal remains	This Study	A7437/T20248
Ancient skeletal remains	This Study	A7437/T20248

**Chemicals, peptides, and recombinant proteins**		

Sodium hypochlorite - made up to 2–3% aqueous with DI water (see below)	Sigma-Aldrich	CAT#1.05614.0000
Acetic Acid (glacial) - made up to 0.1 M using DI water (see below)	Sigma-Aldrich	CAT#A6283
De-ionised water via Labwater system	Laboratory Water Systems, Labwater Range	SKU: L991009

**Software and algorithms**		

	R: A Language and Environment for Statistical Computing. http://www.r-project.org/	
	RStudio: Integrated Development Environment for R. http://www.rstudio.com/	
	OxCal v.4.4.4. Bronk Ramsey, Christopher. “Bayesian Analysis of Radiocarbon Dates.” *Radiocarbon* 51, no. 1 (ed 2009): 337–60. https://doi.org/10.1017/S0033822200033865.	

	Code associated with the work is found in the Mendeley dataset under Vikings_Isotopes_Publication_ Script.R	https://doi.org/10.17632/3pkp8kg6g3.2

**Deposited Data**		

	Mendeley dataset	https://doi.org/10.17632/3pkp8kg6g3.2

### Resource availability

#### Lead contact

Further information and requests for resources and reagents should be directed to and will be fulfilled by the lead contact and corresponding author Lisa Mariann Strand (lisa.strand@ntnu.no).

#### Materials availability

This study did not generate unique reagents; There are restrictions to the availability of reagents due to that the Late Iron and Viking Age skeletal human remains in this study is in its entirety few, therefore considered to be a limited resource. Use of these remains is contingent on approval from the Norwegian National Committee for Research Ethics on Human Remains. The remains are physically stored at Oslo University Hospital.

### Experimental model and subject detail

#### The burials and human remains

Overall, the human remains which encompass this study were excavated in the period from the mid-19th century towards the late 20th century, with an emphasis on the early 20th century (Margaryan et al., 2020). This extended time, stretching from the 19th century union period with Sweden, throughout the duration of the 20th century post-war period which is marked by a relatively high frequency of archaeological examination and excavations of among other Late Iron and Viking Age burials in Norway. The contexts in this study were both located by the coast as well as found on inland locations (Figure 1A in main text), and the ^14^C mixed marine calibration show that the human remains in this study, overall, date from the Late Iron and Viking Ages (Table S3 in the supplementary section).

All work was done in accordance with the relevant ethical guidelines, as approved by the Norwegian National Committee for Research Ethics on Human Remains (2018/364). The biological sex (Margaryan et al., 2020) as well as age at death is described below and summarized in Table S1.

##### Troms county

A4049/Ts3525 Male, aged between 35 and 49. Year of excavation: 1933. A4184/Ts3639 Female, aged between 16 and 20. Year of excavation: 1935.

##### Nordland county

A253/C18558 Male. Aged between 15 and 17 years. Year of excavation: 1889. A642/T5105 Male, aged between 35 and 49. Year of excavation: 1897. A1502/C18035 Male, aged >50. Year of excavation: 1894. A1522/C14554 Male, aged between 20 and 25. Year of excavation: 1889. A3708/T9366 Male, aged between 40 and 50. Year of excavation: C. 1910. A3709/T12578 Female, aged between 35 and 49. Year of excavation. 1922. A4511/Ts4306 Female, aged between 20 and 35. Year of excavation: C.1946. A4512/Ts4306 Male, aged between 20 and 35. Year of excavation: C.1946. A4689/Ts5252 Female, aged between 35 and 49. Year of excavation: 1954. A4691b/Ts5287 Male, aged between 22 and 35. Year of excavation: 1954. A4727/Ts5656 Male, aged between 40 and 50. Year of excavation: 1957. A5195/Ts7659 Male, aged between 10 and 12. Year of excavation: 1965/66. A5317/T20545 Female, aged between 15 and 17. Year of excavation: 1983.

##### Nord-trøndelag county

A3705/T2327 Female, aged between 35 and 49. Year of excavation: 1927. A7437/T20248. Three individual human remains with the same anthropological number and museum number. Two is considered female, one individual unknown. One individual is aged between 35 and 49, one individual is aged between 19 and 20 and one is aged between 1 and 1.5-year-old. Year of excavation: 1981 (NTNU University Museum Collection, 2022b).

##### Sør-trøndelag county

A3699/T13363 Female, aged between 35 and 49. Year of excavation: 1926. A4481/T16298 Male, aged >50. Year of excavation: 1944.

##### Oppland county

A1517/C14690 Male, aged between 35 and 49. Year of excavation: 1889. A1520/C21852 Female, aged between 35 and 49. Year of excavation: 1910. A2808(1), A2808(2) Female and Male, aged between 25-35 and 35–49. Year of excavation: 1868. A3777/C24243 Male, aged between 35 and 49. Year of excavation: 1928. A3778/C24297 Female, aged between 35 and 49. Year of excavation: 1928. A5305/C35586 Male, aged between 35 and 49. Year of excavation: 1981.

##### Hedmark county

A2813/C17564 Male, aged between 55 and 65. Year of excavation: C. 1893. A4005/C25552 Female, aged between 35 and 49. Year of excavation: 1933. A4006/C25720 Female, aged between 35 and 49. Year of excavation: 1933. A4304/C26737 Male, aged between 20 and 25. Year of excavation: 1938. A4460/C27338 Male, aged between 35 and 49. Year of excavation: C. 1943.

##### Telemark county

A1645/C21794 Female, aged between 55 and 65. Year of excavation:1915. A3697/C23941 Male, aged between 40 and 50. Year of excavation: 1926.

#### Materials

30 (see Table S3) of approximately 140 known human remains considered to be from the Late Iron and Viking Age (The Schreiner Collection database, 2022a) were analyzed for isotopic signatures associated with mobility (δ^18^O) (N = 23) and diet (δ^13^C, δ^15^N) (N = 22). The skeletal material in question is unique, and we were constrained by ethical regulations to mostly use residual material remaining from analyses of ancient DNA to avoid further destruction of human remains through analysis (Margaryan et al., 2020).

Such a low sample size indicates a biased representation of the human skeleton as well as grave selection representativeness, both by the overall representation of the human skeleton, and in the selection of the inhumation burials (DeWitte and Stojanowski, 2015; Wood et al., 1992). However, efforts were made to achieve equal representation of both biological sex and age groupings (Table S1) and a broad regional distribution (Figure 1A) by the investigators on the genetic study (Margaryan et al., 2020). Likewise, skeletal taphonomy and considerations of ethical regulations by the Norwegian national committee for research ethics on human remains which manages regional selection with reference LOJ (2018/364), formed part of the sampling strategy. Such concerns surpass parameters such as even distribution.

The intentions to strive for equal representation of both biological sex and age groupings as well as regional distribution (Table S1and Figure 1A) had previously guided the genetic study (Margaryan et al., 2020). Representation of human remains from the Late Iron and Viking Age in Norway are, due to preservation conditions, varied. Further, as there are few human remains from this period which are available for biomolecular research, efforts to re-use already sampled skeletal elements from prior projects were in focus, which is the cause for the uneven representation per sampled individual. Nevertheless, radiocarbon dating calibrated for the marine reservoir effect creates an opportunity to separate events such as individual mobility which, in consequence, creates opportunities to identify mobility and diet throughout the Norwegian Late Iron and Viking Age, creating the space to discuss levels of gendered dimensions during this period.

### Method details

#### Stable isotope analyses and radiocarbon dating

##### Carbonate

Tooth enamel samples were drilled from bones, and premolar and molar teeth, essentially as described (Miller et al., 2018). The samples were drilled using a rig set up with a Dremel 4000 and diamond-tipped drills at low speed (power level 2 out of 5) to avoid overheating. Enamel and osteologial material were collected as a powder over aluminum foil, which was changed after every drilled sample. The powder were then transfered into a 2.0 mL micro-centrifuge tube. After each sample, the equipment as well as the workspace were cleaned according to protocol. Briefly, the equipment was first cleaned with 0.5 M hydroclorid acid, and thereafter wiped down with methanol and left to dry. The equipment was also sprayed by compressed air. The workspace was then cleaned by the use of a brush and dustpan, as well as wiped down with methanol.

Tooth enamel powder was prepared for stable isotope analysis of bioapatite (carbonate) following the Balasse method, using Dorothy Garrod Laboratory protocols (Balasse et al., 2002; Leggett et al., 2021). Enamel powder and laboratory standards were treated with 0.1 mL/mg of powder of 2–3% NaOCl(aq.) for 24 h at 4°C and rinsed, vortexed and centrifuged five times in distilled water. To remove secondary carbonates samples were then treated with 0.1 mL/mg of powder of 0.1 M acetic acid(aq.) for 4 h at room temperature, then rinsed, vortexed and centrifuged five times with distilled water. Once all liquid is removed samples were frozen at −20°C for 1 h and transferred to −80°C for a minimum of 2 h, and then lyophilised between 2 and 4 h. The resulting powder was weighed to establish loss of enamel during pre-treatment (40–50% loss in sample weight is expected). Between 2 and 4 mg of treated enamel powder was transferred into glass vials sealed with a screw cap holding a septa and PCTE washer to create a vacuum seal. The vacuum sealed glass vials containing the enamel were reacted with 10% orthophosphoric acid at 90°C using a Gas Bench II coupled to a Delta V mass spectrometer for isotopic analysis. The enamel carbonate isotope values are reported in units permille with reference to the VPDB standard calibrated through the NBS19 standard for carbon δ^13^C_VPDB_ = [(^13/11^C_sample_/^13/12^C_VPDB_)-1]x 1000, and with reference to VSMOW (Vienna Standard Mean Ocean Water) for δ^18^O using internal standards such that δ^18^O_VSMOW_ = [(^18/16^O_sample_/^18/16^C_STANDARD_)-1]x 1000 put in (Coplen, 1994, 2011; Hoefs, 2018). Analytical error for the carbonate samples is ±0.08‰ for δ^13^C and ±0.10‰ for δ^18^O.

δ^18^O_MAP_ values were obtained for each site using the Online Isotopes in Precipitation Calculator (OIPC version 3.2) (Bowen and Revenaugh, 2003; Leggett et al., 2021; Lightfoot and O’Connell, 2016). Site elevations were obtained for these calculations using Google EarthPro (Google Earth pro, 2019) As per Leggett et al. (2021) (Leggett et al., 2021) δ^18^O_carbonate_ values were then converted to δ^18^O_phosphate_, δ^18^O_dw_ and Δ^18^O_dw-MAP_ values were calculated as an offset between the burial site δ^18^O_MAP_ values and the individuals’ tooth enamel δ^18^O_dw_ values as a rough measure of the extent to which they are local or non-local. Due to propagated error in δ^18^O conversions Δ^18^O_dw-MAP_ values within ±2‰ must be considered “local”, those beyond −2‰ are considered migrants from colder areas than the burial site, and those beyond +2‰ could be migrants but must have “brewing and stewing” factored into their interpretation (Brettell et al., 2012; Bowen, 2019; Leggett, 2021a; Leggett et al., 2021; Pederzani and Britton, 2019).

Additionally, where ^87^Sr/^86^Sr data was available for individuals (Naumann, 2014; Price and Naumann, 2014), this was added to the δ^18^O data to run single and dual-isotope probabilistic geographic assignments for 29 individuals (Figures 6 and 7). These models are outputted as maps displaying the probabilistic geographical scope of an individual’s isotopic data, given known and modeled baseline isotopic data. The darker the green on the map the better the isotopic match between the tooth data and the climate and geological isotopic data, and the higher the likelihood that the person might have spent time in that area during the period of tooth formation. This is meant to aid archaeological interpretations, and whilst it can rule out certain areas, caution must be used when interpreting possible regions highlighted, and “brewing and stewing” effects should still be considered. Modifications were made to the equations used by Bataille et al., (2021) (Bataille et al., 2021) – namely the use of the Chenery conversion equations for drinking water, as we felt they were more conservative, and a change to their code for plotting site geographic coordinates as their original code did not work as published (Bataille et al., 2021; Chenery et al., 2012; Colleter et al., 2021). These and all other graphs of isotopic data produced here were produced using free and Open Source R (version 4.0.4) and RStudio (version 1.4.1106) (R Development Core Team, 2021b; RStudio Team, 2021c). All data spreadsheets and R code for these models are available here as supplemental information. Full details of packages and code used are supplied in the R code.

#### Collagen for stable isotopes and radiocarbon dating

The same collagen extraction process was used for both radiocarbon determination and analysis by isotope ratio mass spectrometry (IRMS). Extraction was undertaken using a modified Longin method as per the protocols outlined in Seiler et al. (2019) at the Trondheim Radiocarbon Laboratory (Longin, 1971; Seiler et al., 2019). Bone samples were first crushed into fine pieces to speed up the chemical reactions. Samples were ultrasonicated in H_2_O, followed by acetone to remove lipids. Then samples were demineralized in 3.6% HCl at room temperature until the pH of the solution stabilized at <1. Samples were then washed with deionized pH3 water and 0.5% NaOH was added for 4 h at room temperature to dissolve any humic acids. The residues were then acidified again with 3.6% HCl to remove any atmospheric CO_2_, washed again with pH3 water and hydrolyzed at 70°C overnight. Finally, the gelatinized samples were filtered whilst hot through a pre-combusted quartz filter (Merck Millipore, AQFA04700) and freeze-dried. The freeze-dried samples were then weighed out into aliquots of approximately 0.75 mg for IRMS.

For δ^13^C and δ^15^N measurements the collagen was combusted in a Flash 200 elemental analyser coupled to a Delta V Plus mass spectrometer in continuous flow mode through the ConFlow IV interface (ThermoScientific). All samples were measured in triplicate where possible and the average of these aliquots is reported here. The collagen preservation was good across the samples used for both radiocarbon and isotopic analysis, with all samples meeting the established standards for collagen preservation (Van Klinken, 1999). For an overview over the C:N ratio and collagen yields for the samples used in this study, see Tables S7 and S8 in the supplementary section.

Stable isotope concentrations are measured as the ratio of the heavier to the lighter isotope. These values are reported relative to an internationally defined scale - VPDB (δ^13^C) and AIR (δ^15^N). Where δ^15^N_AIR_ = [(^15/14^N_sample_/^15/14^N_AIR_)-1], and δ^13^C_VPDB_ = [(^13/11^C_sample_/^13/12^C_VPDB_)-1] (Brown and Brown, 2011; Coplen, 2011; Hoefs, 2018; Price and Burton, 2012). Analytical error was 0.10‰ for δ^15^N and 0.12‰ for δ^13^C measurements. Internal laboratory standards were used to determine analytical error with values between −1.0‰ and 15.0‰ for δ^15^N, and −41.0‰ and −16.5‰ for δ^13^C. Internal reference gases (N_2_ ref 1, N_2_ ref 2, sample N_2_, sample CO_2_, CO_2_ ref 1, CO_2_ ref 2) calibrated to an international standard (IAEA-600) were also used to determine the stability of the system and to normalize the samples. The resulting ratios are δ^15^N = −0.81‰ for N_2_ and δ^13^C = −4.02‰ for CO_2_. Including the uncertainties of the reference gases, the absolute measurement uncertainty for the measurement becomes 0.22‰ for δ^15^N and 0.13‰ for δ^13^C.

For radiocarbon determination the collagen samples were then combusted and reduced to produce homogeneous graphite for AMS (accelerator mass spectrometry) measurement. Two different reduction lines are used at the Trondheim lab, all samples reported here underwent the Trondheim Oxidation and Reduction-system (TOR) (Seiler et al., 2019). After reduction, samples were measured on the 1 MV AMS system (High Voltage Engineering Europe B.V.). Details of the system and laboratory standards can be found in the laboratory’s radiocarbon status report (Seiler et al., 2019).

^14^C is unequally distributed in the biosphere, and, despite carbon exchanging in dynamic equilibrium with surface ocean waters, there is, due to the large area of the global ocean, a carbon reservoir with a slow rate of mixing, giving an offset between oceanic and atmospheric ^14^C which averages at approximately 400 years in sub-tropical areas to up to 1000 years closer to the poles (Heaton et al., 2020; Jarman et al., 2018). This reservoir effect is passed onto marine plants and animals and into consumers of marine resources. Therefore, humans who eat a significant amount of marine resources will have radiocarbon determinations that look older than they are. This effect has been demonstrated for Mesolithic and medieval populations in Scotland and the Viking Great Army burials at Repton in England (Ascough et al., 2017; Jarman et al., 2018; Russell et al., 2010). Freshwater resources are known to have similar effects, but applying corrections is currently extremely difficult due to the intricacies of carbon cycling in freshwater ecosystems, and so this has not been attempted here (Dury et al., 2018; Ervynck et al., 2018; Guiry, 2019). Therefore, ^14^C dates from our collagen samples were re-calibrated from those provided by the Trondheim lab to take into account the MRE as per Jarman et al. (2018) (Jarman et al., 2018). Only collagen samples were re-calibrated for MRE; dates from carbonate were not re-calibrated – this was because of major methodological issues and a lack of consensus in how to calculate MRE corrections for human carbonate. We used the published ΔR values for the North Norwegian Sea 17 ± 36 for the individuals buried in Nordland and Troms counties (Dury et al., 2018) and −33 ± 27 for southern Norway, respectively (Heier-Nielsen et al., 1995; Jarman et al., 2018).

To estimate the proportion of marine foods in each individual’s diet we used the stable isotope measurements, preferencing IRMS δ^13^C_collagen_ values over AMS data where available. This can be done in a variety of ways, using various mixing models and software (Parnell et al., 2013; Phillips, 2012). For simplicity we here followed Jarman et al. (2018) (Jarman et al., 2018) and used a linear interpolation from isotopic end members, using only δ^13^C_collagen_ values since δ^15^N_collagen_ values vary geographically and are highly sensitive to local environmental baseline differences, physiological differences (e.g. stress, breastfeeding etc.) and can be obscured more readily by freshwater dietary input (Cook et al., 2015; Dury et al., 2018; Fuller et al., 2005; Guiry, 2019; Jarman et al., 2018; Pollard et al., 2012; Russell et al., 2010; Szpak, 2014). The equation used to calculate the fraction of marine dietary protein (f_m_) for the given individual *n* is:

$$f_{m=\frac{\delta^{13}C_{n}-\delta^{13}C_{terr}}{\delta^{13}C_{mar}-\delta^{13}C_{terr}}}$$

Where δ^13^C_n_ is the isotopic measurement for the sample *n*, and δ^13^C_mar_ is the fully marine isotopic end member and δ^13^C_terr_ is the fully terrestrial end member. End members were chosen from available published data from contemporary Norway in Leggett et al. (2021) (Leggett et al., 2021). The terrestrial end member was calculated from domestic and wild herbivores from Viking Age contexts in Norway averaging δ^13^C_coll_ values of −22.1‰ (Leggett et al., 2021; Naumann et al., 2014a; Van der Sluis et al., 2016); the marine end member is a marine fish (ling) from Viking Age layers at Stavanger with a δ^13^C_coll_ value of −12.6‰ and was the most ^13^C enriched of all the available contemporary marine faunal values (Leggett et al., 2021; Van der Sluis et al., 2016). Since there are no known C_4_ food sources known to be available in northern Europe for the period, less negative δ^13^C_coll_ values in this study should therefore reflect marine resource consumption (Hakenbeck et al., 2010; Jarman et al., 2018; Kosiba et al., 2007; Leggett, 2021a). A generalized uncertainty of 10% was added to the estimates of proportion of marine intake for use in OxCal, at the expense of precision, to account for the propagation of error and to vary levels of knowable and unknowable uncertainty by using mixing models, the choice of end members not being ideal, and migration of the individuals analyzed (see below) (Hadley and Richards, 2016; Jarman et al., 2018).OxCal v.4.4.4 was used to calibrate the radiocarbon determinations using the IntCal20 atmospheric curve and the Marine20 marine calibration curve given the ΔR value and marine proportions calculations for each sample detailed above Bronk Ramsey 2021 internet (Kosiba et al., 2007; Price and Burton, 2012).

### Quantification and statistical analysis

In Figures 2, 3, 4, and 5, each symbol represents the values associated with measurements of skeletal material from Viking Age graves (n = 1 per sample). Since we have only sampled one bone or tooth per individual, n can also be related to individual graves identified through Archaeological ID and Museum-ID in Supplementary Tables and supplementary Excel file. In Figure 4, we report the averaged values of Δ^18^O_dw-MAP_ in the form of a violin plot, with interquartile ranges indicated. All data can be found in the Supplementary Tables and supplemental excel file. We have not performed statistical tests to compare between groups, and hence, no assumptions on the statistical approach have been made.

## Data Availability

• Data associated with the publication is found in the Mendeley dataset with the https://doi.org/10.17632/3pkp8kg6g3.2. • Code associated with the publication is found in the Mendeley dataset under Vikings_Isotopes_Publication_ Script.R. https://doi.org/10.17632/3pkp8kg6g3.2. • Other items: there are no other items associated with the publication.

## Supporting Citations

The following reference appears in the supplemental information: Vike, 2016.

## Supplemental information

Document S1. Figure S1 and Tables S1–S8

Data S1. Excel file relatable to Figures 1–7 in the main text and Tables S1–S8 in the supplementary contains all data pertaining to this article The file relates to Figures 1A, 2A, 2B, 3, 4, 5, 6, and 7 in the main text, as well as Tables S1–S8 in the supplementary section.

## References

[bib1] Ambrose S.H., Norr L. (1993). Prehistoric Human Bone.

[bib2] Androshchuk F. (2014).

[bib3] Ascough P.L., Church M.J., Cook G.T. (2017). Marine radiocarbon reservoir effects for the Mesolithic and Medieval periods in the western isles of Scotland. Radiocarbon.

[bib4] Balasse M., Ambrose S.H., Smith A.B., Price T. (2002). The seasonal mobility model for prehistoric herders in the south-western cape of South Africa assessed by isotopic analysis of sheep tooth enamel. J. Archaeol. Sci..

[bib5] Barrett J.H. (2016). Cod and Herring: The Archaeology and History of Medieval Sea Fishing.

[bib6] Bataille C.P., Jaouen K., Milano S., Trost M., Steinbrenner S., Crubézy É., Colleter R. (2021). Triple sulfur-oxygen-strontium isotopes probabilistic geographic assignment of archaeological remains using a novel sulfur isoscape of western Europe. PLoS One.

[bib7] Beaumont J., Montgomery J., Buckberry J., Jay M. (2015). Infant mortality and isotopic complexity: new approaches to stress, maternal health, and weaning: infant Mortality and Isotopic Complexity. Am. J. Phys. Anthropol..

[bib8] Beaumont J., Atkins E.C., Buckberry J., Haydock H., Horne P., Howcroft R., Mackenzie K., Montgomery J. (2018). Comparing apples and oranges: why infant bone collagen may not reflect dietary intake in the same way as dentine collagen. Am. J. Phys. Anthropol..

[bib9] Beaumont J. (2020). The whole tooth and nothing but the tooth: or why temporal resolution of bone collagen may Be unreliable. Archaeometry.

[bib10] Berglund I.B. (1996). Tjøtta-riket: en arkeologisk undersøkelse av maktforhold og sentrumsdannelser på Helgelandskysten fra Kr. f. til 1700 e. Kr. UNIT, Vitenskapsmuseet, Fakultet for arkeologi og kulturhistorie.

[bib11] Bocherens H., Drucker D. (2003). Trophic level isotopic enrichment of carbon and nitrogen in bone collagen: case studies from recent and ancient terrestrial ecosystems. Int. J. Osteoarchaeol..

[bib12] Bogaard A., Heaton T.H.E., Poulton P., Merbach I. (2007). The impact of manuring on nitrogen isotope ratios in cereals: archaeological implications for reconstruction of diet and crop management practices. J. Archaeol. Sci..

[bib13] Bowen G.J., Revenaugh J. (2003). Interpolating the isotopic composition of modern meteoric precipitation. Water Resour. Res..

[bib14] Bowen G.J. (2019). The online isotopes in precipitation calculator, version 3.2. http://www.waterisotopes.org.

[bib15] Bratrein H.D. (2018).

[bib16] Brettell R., Montgomery J., Evans J. (2012). Brewing and stewing: the effect of culturally mediated behaviour on the oxygen isotope composition of ingested fluids and the implications for human provenance studies. J. Anal. At. Spectrom..

[bib17] Brown T.A., Brown K. (2011).

[bib18] Burt N.M. (2013). Stable isotope ratio analysis of breastfeeding and weaning practices of children from medieval Fishergate House York, UK. Am. J. Phys. Anthropol..

[bib19] Chenery C.A., Pashley V., Lamb A.L., Sloane H.J., Evans J.A. (2012). The oxygen isotope relationship between the phosphate and structural carbonate fractions of human bioapatite: relationship between phosphate and structural carbonate δ18O in human enamel. Rapid Commun. Mass Spectrom..

[bib20] Chenery C.A., Evans J.A., Score D., Boyle A., Chenery S.R. (2014). A boat load of Vikings?. J. N. Atl..

[bib21] Chisholm B.S., Nelson D.E., Schwarcz H.P. (1982). Stable-carbon isotope ratios as a measure of marine versus terrestrial protein in ancient diets [Dietary analysis of prehistoric people, Canada’s Pacific coast]. Science.

[bib22] Christys A. (2015).

[bib23] Clementz M.T., Fox-Dobbs K., Wheatley P.V., Koch P.L., Doak D.F. (2009). Revisiting old bones: coupled carbon isotope analysis of bioapatite and collagen as an ecological and palaeoecological tool. Geol. J..

[bib24] Colleter R., Bataille C.P., Dabernat H., Pichot D., Hamon P., Duchesne S., Labaune-Jean F., Jean S., Le Cloirec G., Milano S. (2021). The last battle of Anne of Brittany: solving mass grave through an interdisciplinary approach (paleopathology, biological anthropology, history, multiple isotopes and radiocarbon dating). PLoS One.

[bib25] Cooijmans C. (2020).

[bib26] Cook G.T., Ascough P.L., Bonsall C., Hamilton W.D., Russell N., Sayle K.L., Scott E.M., Bownes J.M. (2015). Best practice methodology for 14C calibration of marine and mixed terrestrial/marine samples. Quat. Geochronol..

[bib27] Coplen T.B. (1994). Reporting of stable hydrogen, carbon, and oxygen isotopic abundances (technical report). Pure Appl. Chem..

[bib28] Coplen T.B. (2011). Guidelines and recommended terms for expression of stable-isotope-ratio and gas-ratio measurement results. Rapid Commun. Mass Spectrom..

[bib29] DeWitte S.N., Stojanowski C.M. (2015). The osteological paradox 20 years later: past perspectives, future directions. J. Archaeol. Res..

[bib30] Dumwille D.N. (2008). the Viking World.

[bib31] Dury J.P.R., Eriksson G., Fjellström M., Wallerström T., Lidén K. (2018). Consideration of freshwater and multiple marine reservoir effects: dating of individuals with mixed diets from Northern Sweden. Radiocarbon.

[bib32] Ervynck A., Boudin M., Van Neer W. (2018). Assessing the radiocarbon freshwater reservoir effect for a northwest-european river system (the schelde basin, Belgium). Radiocarbon.

[bib33] Fuller B.T., Fuller J.L., Sage N.E., Harris D.A., O’Connell T.C., Hedges R.E.M. (2005). Nitrogen balance and δ15N: why you’re not what you eat during nutritional stress. Rapid Commun. Mass Spectrom..

[bib34] Fuller B.T., Molleson T.I., Harris D.A., Gilmour L.T., Hedges R.E.M. (2006). Isotopic evidence for breastfeeding and possible adult dietary differences from late/sub-roman britain. Am. J. Phys. Anthropol..

[bib35] Gardeła L. (2021).

[bib36] Glørstad Z.T. (2012). Sign of the times? The transfer and transformation of penannular brooches in viking-age Norway. Norweg. Archaeol. Rev..

[bib37] Google (2019) Google Earth pro.Digital. GoogleReference home page for NTNU University Museum Collection. https://collections.vm.ntnu.no/.

[bib38] Guiry E. (2019). Complexities of stable carbon and nitrogen isotope biogeochemistry in ancient freshwater ecosystems: implications for the study of past subsistence and environmental change. Front. Ecol. Evol..

[bib39] Hadley D.M., Hemer K.A. (2011). Microcosms of migration: children and early medieval population movement. Child. Past.

[bib40] Hadley D.M., Richards J.D. (2016). The winter camp of the viking great Army, AD 872–3, torksey, lincolnshire. Antiq. J..

[bib41] Hakenbeck S., McManus E., Geisler H., Grupe G., O’Connell T. (2010). Diet and mobility in Early Medieval Bavaria: a study of carbon and nitrogen stable isotopes. Am. J. Phys. Anthropol..

[bib42] Hamre S.S., Daux V. (2016). Stable oxygen isotope evidence for mobility in medieval and post-medieval Trondheim, Norway. J. Archaeol. Sci. Rep..

[bib43] Haydock H., Clarke L., Craig-Atkins E., Howcroft R., Buckberry J. (2013). Weaning at Anglo-Saxon raunds: implications for changing breastfeeding practice in britain over two millennia. Am. J. Phys. Anthropol..

[bib44] Heaton T.J., Köhler P., Butzin M., Bard E., Reimer R.W., Austin W.E.N., Bronk Ramsey C., Grootes P.M., Hughen K.A., Kromer B. (2020). Marine20—the marine radiocarbon age calibration curve (0–55, 000 cal BP). Radiocarbon.

[bib45] Hedenstierna-Jonson C., Kjellström A., Zachrisson T., Krzewińska M., Sobrado V., Price N., Günther T., Jakobsson M., Götherström A., Storå J. (2017). A female Viking warrior confirmed by genomics. Am. J. Phys. Anthropol..

[bib46] Hedges R.E., Reynard L.M. (2007). Nitrogen isotopes and the trophic level of humans in archaeology. J. Archaeol. Sci..

[bib47] Heen-Pettersen A.M. (2019). The earliest wave of Viking activity? The Norwegian evidence revisited. Eur. J. Archaeol..

[bib48] Heier-Nielsen S., Heinemeier J., Nielsen H.L., Rud N. (1995). Recent reservoir ages for Danish fjords and marine waters. Radiocarbon.

[bib49] Hoefs J. (2018).

[bib50] Jarman C.L., Biddle M., Higham T., Bronk Ramsey C. (2018). The viking great Army in England: new dates from the Repton charnel. Antiquity.

[bib51] Kendall E., Millard A., Beaumont J. (2021). The “weanling’s dilemma” revisited: evolving bodies of evidence and the problem of infant paleodietary interpretation. Am. J. Phys. Anthropol..

[bib52] Kosiba S.B., Tykot R.H., Carlsson D. (2007). Stable isotopes as indicators of change in the food procurement and food preference of Viking Age and Early Christian populations on Gotland (Sweden). J. Anthropol. Archaeol..

[bib53] Krzewińska M., Bjørnstad G., Skoglund P., Olason P.I., Bill J., Götherström A., Hagelberg E. (2015). Mitochondrial DNA variation in the Viking age population of Norway. Philos. Trans. R. Soc. Lond. B Biol. Sci..

[bib54] Kusaka S., Uno K.T., Nakano T., Nakatsukasa M., Cerling T.E. (2015). Carbon isotope ratios of human tooth enamel record the evidence of terrestrial resource consumption during the Jomon period, Japan: carbon isotope ratios of Jomon enamel. Am. J. Phys. Anthropol..

[bib55] Kusaka S. (2019). Stable isotope analysis of human bone hydroxyapatite and collagen for the reconstruction of dietary patterns of hunter-gatherers from Jomon populations. Int. J. Osteoarchaeol..

[bib56] Leggett S., Rose A., Praet E., Le Roux P. (2021). Multi-tissue and multi-isotope (δ13C, δ15N, δ18O and 87/86Sr) data for early medieval human and animal palaeoecology.

[bib57] Leggett S. (2021).

[bib58] Leggett S. (2021). Tackling western European dietary transitions in the first millennium AD: a hierarchical meta-analytical approach.

[bib59] Lightfoot E., O’Connell T.C. (2016). On the use of biomineral oxygen isotope data to identify human migrants in the archaeological record: intra-sample variation, statistical methods and geographical considerations. PLoS One.

[bib60] Lightfoot E., Ustunkaya M.C., Przelomska N., O’Connell T.C., Hunt H.V., Jones M.K., Petrie C.A. (2020). Carbon and nitrogen isotopic variability in foxtail millet (Setaria italica) with watering regime. Rapid Commun. Mass Spectrom..

[bib61] Løkka N. (2014). Kvinner i Vikingtid.

[bib62] Longin R. (1971). New method of collagen extraction for radiocarbon dating. Nature.

[bib63] Margaryan A., Lawson D.J., Sikora M., Racimo F., Rasmussen S., Moltke I., Cassidy L.M., Jørsboe E., Ingason A., Pedersen M.W. (2020). Population genomics of the Viking world. Nature.

[bib64] Mazet-Harhoff L. (2010). Viking Trade and Settlement in Continental Western Europe.

[bib65] Mikkelsen E. (2019).

[bib66] Miller A.V., Fernandes R., Janzen A., Nayak A., Swift J., Zech J., Boivin N., Roberts P. (2018). Sampling and pretreatment of tooth enamel carbonate for stable carbon and oxygen isotope analysis. JoVE.

[bib67] Moen M. (2010).

[bib68] Moilanen U., Kirkinen T., Saari N.-J., Rohrlach A.B., Krause J., Onkamo P., Salmela E. (2021). A woman with a sword?–weapon grave at Suontaka Vesitorninmäki, Finland. Eur. J. Archaeol..

[bib69] Mühlemann B., Vinner L., Margaryan A., Wilhelmson H., de la Fuente Castro C., Allentoft M.E., de Barros Damgaard P., Hansen A.J., Holtsmark Nielsen S., Strand L.M. (2020). Diverse variola virus (smallpox) strains were widespread in northern Europe in the Viking Age. Science.

[bib70] Naumann E., Price T.D., Richards M.P. (2014). Changes in dietary practices and social organization during the pivotal Late Iron Age period in Norway (AD 550–1030): isotope analyses of Merovingian and Viking Age human remains. Am. J. Phys. Anthropol..

[bib71] Naumann E., Krzewińska M., Götherström A., Eriksson G. (2014). Slaves as burial gifts in Viking Age Norway? Evidence from stable isotope and ancient DNA analyses. J. Archaeol. Sci..

[bib72] Naumann E., Glørstad A.Z.T., Breiby M.P., Mills R.D., Fullagar P.D. (2019). Who were the first urban settlers of Oslo? A discussion of early medieval urbanization based on isotopic analyses of human remains. Archaeometry.

[bib73] Naumann E. (2014).

[bib74] Nielssen A., Barrett J., Orton D. (2016). Cod and Herring: The Archaeology and History of Medieval Sea Fishing.

[bib75] (2022b). NTNU University Museum Collection. https://collections.vm.ntnu.no.

[bib76] Pantmann P. (2014). Kvinner i Vikingtid.

[bib77] Parnell A.C., Phillips D.L., Bearhop S., Semmens B.X., Ward E.J., Moore J.W., Jackson A.L., Grey J., Kelly D.J., Inger R. (2013). Bayesian stable isotope mixing models. Environmetrics.

[bib78] Pederzani S., Britton K. (2019). Oxygen isotopes in bioarchaeology: principles and applications, challenges and opportunities. Earth Sci. Rev..

[bib79] Pellegrini M., Pouncett J., Jay M., Pearson M.P., Richards M.P. (2016). Tooth enamel oxygen “isoscapes” show a high degree of human mobility in prehistoric Britain. Sci. Rep..

[bib80] Petersen J. (1919).

[bib81] Phillips D.L. (2012). Converting isotope values to diet composition: the use of mixing models. J. Mammal..

[bib82] Pollard A.M., Ditchfield P., Piva E., Wallis S., Falys C., Ford S. (2012). ‘Sprouting like cockle amongst the wheat’: the St Brice’s Day massacre and the isotopic analysis of human bones from St John’s College, Oxford. Oxf. J. Archaeol..

[bib83] Price T., Burton J. (2012).

[bib84] Price T.D., Naumann E. (2014). The peopling of the north atlantic: isotopic results from Norway. J. N. Atl..

[bib85] Price N. (2008). The Viking Way.

[bib86] R Development Core Team (2021) R: a language and environment for statistical computing. R. http://www.r-project.org/

[bib87] Raffield B. (2016). Bands of brothers: a re-appraisal of the viking great Army and its implications for the scandinavian colonization of England. Early Mediev. Eur..

[bib88] Raffield B. (2019). Playing Vikings: militarism, hegemonic masculinities, and childhood enculturation in Viking Age Scandinavia. Curr. Anthropol..

[bib89] Renaud J. (2008). The Viking World.

[bib90] Reynard L.M., Tuross N. (2015). The known, the unknown and the unknowable: weaning times fromarchaeological bones using nitrogen isotope ratios. J. Archaeol. Sci..

[bib91] RStudio Team (2021).

[bib92] Russell N., Cook G.T., Ascough P.L., Dugmore A.J. (2010). Spatial variation in the marine radiocarbon reservoir effect throughout the Scottish post-Roman to Late Medieval period: north Sea values (500–1350 BP). Radiocarbon.

[bib93] Scheid R.C. (2007).

[bib94] Seiler M., Grootes P.M., Haarsaker J., Lélu S., Rzadeczka-Juga I., Stene S., Svarva H., Thun T., Værnes E., Nadeau M.-J. (2019). Status report of the Trondheim radiocarbon laboratory. Radiocarbon.

[bib95] Sheehan J. (2008). The longphort in Viking-age Ireland. Acta Archaeol..

[bib96] Sjøvold T. (1962).

[bib97] Sjøvold T. (1974).

[bib98] Spangen M. (2005).

[bib99] Stalsberg A. (1991). Social Approaches to Viking Studies.

[bib100] Storli I. (2018).

[bib101] Stylegar F.-A. (2009). Den Urbane Underskog.

[bib102] Stylegar F.-A. (2015). Scandinavia and the Balkans: Cultural Interactions with Byzantium and Eastern Europe in the First Millennium AD.

[bib103] Szpak P. (2014). Complexities of nitrogen isotope biochemistry in plant-soil systems: implications for the study of ancient agricultural and animal management practices. Front. Plant Sci..

[bib104] Tauber H. (1981). 13C evidence for dietary habits of prehistoric man in Denmark. Nature.

[bib105] The Schreiner Collection database (2022). https://www.med.uio.no/imb/english/research/about/schreiner-collection/.

[bib106] Van der Sluis L.G., Hollund H.I., Kars H., Sandvik P.U., Denham S.D. (2016). A palaeodietary investigation of a multi-period churchyard in Stavanger, Norway, using stable isotope analysis (C, N, H, S) on bone collagen. J. Archaeol. Sci. Rep..

[bib107] Van Klinken G.J. (1999). Bone collagen quality indicators for palaeodietary and radiocarbon measurements. J. Archaeol. Sci..

[bib108] Verwers W. (2010). Viking Trade and Settlement in Continental Western Europe.

[bib109] Vike V. (2016). Viking - Norsk Arkeologisk Årbok.

[bib110] Wood J.W., Milner G.R., Harpending H.C., Weiss K.M., Cohen M.N., Eisenberg L.E., Hutchinson D.L., Jankauskas R., Cesnys G., Katzenberg M.A. (1992). The osteological paradox: problems of inferring prehistoric health from skeletal samples [and comments and reply]. Curr. Anthropol..

