# Supplementary material for: Multi-isotope variation reveals social complexity in Viking Age Norway
Source: iScience. 2022 Sep 29;25(10):105225. doi: 10.1016/j.isci.2022.105225 (PMC9579023; doi:10.1016/j.isci.2022.105225)
Supplement: Document S1. Figure S1 and Tables S1–S8 [file mmc1.pdf]

iScience, Volume *25*

Supplemental information

Multi-isotope variation reveals social complexity in Viking Age Norway

Lisa Mariann Strand, Sam Leggett, and Birgitte Skar

Supplementary information

Figure S1: Mixed marine radiocarbon calibrations made by Sam Leggett (OxCal v4.4.4, IntCal20). Figure S1 relate to Figure 1A and Figure 4.

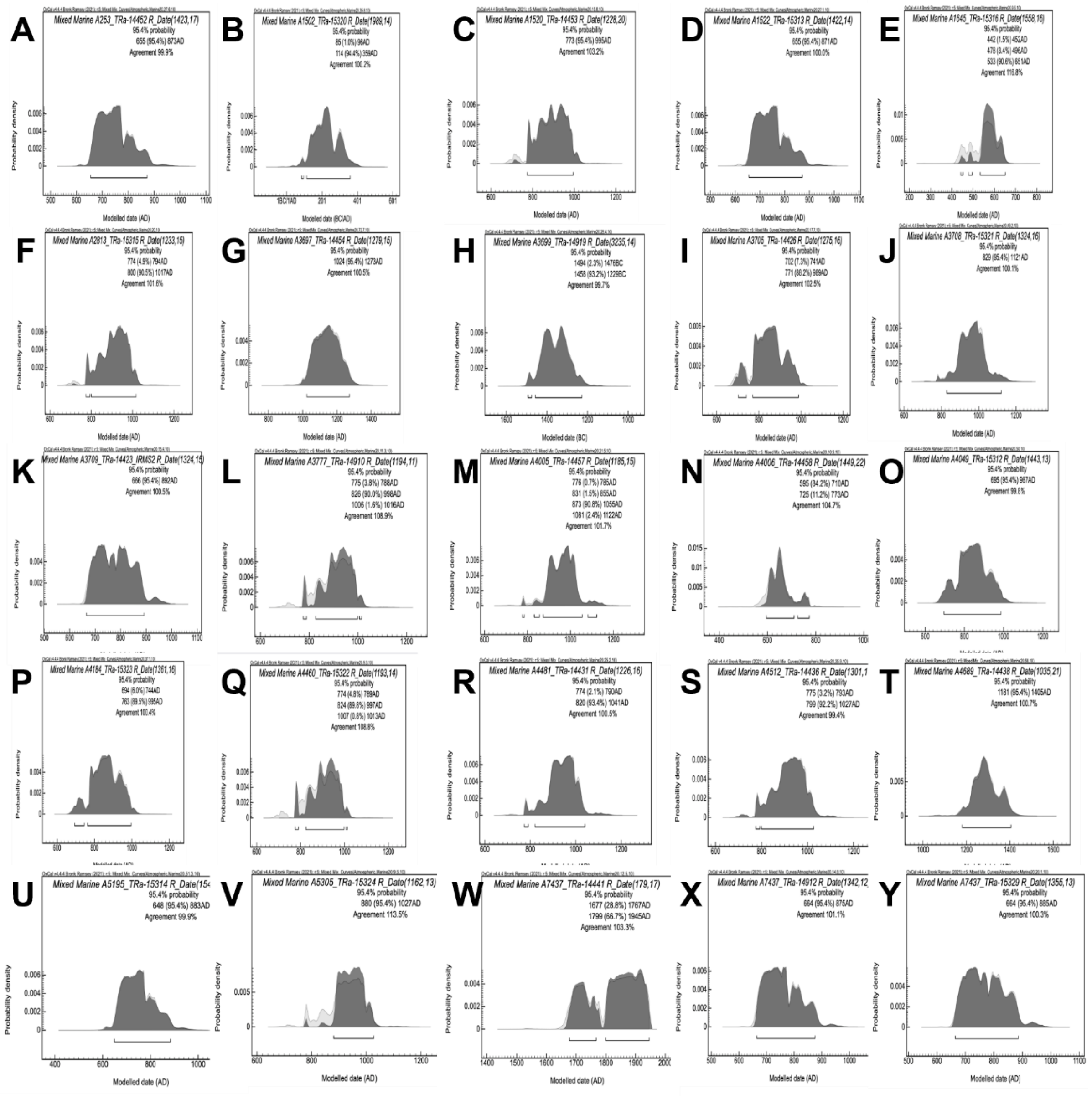

Table S1 – overview of basic information about the human remains which is part of this research. Abbrevation “A-id” is anthropological identification number, “M-id” is museum number, “G-sex” is genetic sex (1), “O-sex” is osteological sex. Table S1 relates to figure 1A, 2B, 3,4 and 5.

A-id	M-id	G-sex sex	O-sex	Age at death	County	Farm
A253	C18558	Male		15-17	Nordland	Ytterstad
A642	T5105	Male		35-49	Nordland	Leines

A1502	C18035	Male		>50	Nordland	Steigen Øvre
A1517	C14690	Male		35-49	Oppland	Nedre Hjeltar
A1520	C21852	Female		35-49	Oppland	Hov
A1522	C14554	Male		20-25	Nordland	Rønvik Øvre
A1645	C21794	Female		55-65	Telemark	Særen
A2808(1)	C4438	Female		25-35	Oppland	Nedre Hov
A2808(2)	C4438	Male		35-49	Oppland	Nedre Hov
A2813	C17564	Male		55-65	Hedmark	Tommelstad
A3697	C23941	Male		40-50	Telemark	Lagmannsgårdshøyden
A3699	T13363	Female		35-49	Sør-Trøndelag	Melem
A3705	T2327	Female		35-49	Nord-Trøndelag	Kil Søndre
A3708	T9366	Male		40-50	Nordland	Tommeidet
A3709	T12578	Female		35-49	Nordland	Løkta
A3777	C24243	Male		35-49	Oppland	Velo Nordre
A3778	C24297	Female		35-49	Oppland	Velo Nordre
A4005	C25552	Female		35-49	Hedmark	Breidablikk
A4006	C25720	Female		35-49	Hedmark	Mæhlum
A4049	Ts3525	Male		35-49	Troms	Bø
A4184	Ts3639	Female		16-20	Troms	Tussøy
A4304	C26737	Male		20-25	Hedmark	Arnestad Lille
A4460	C27338	Male		35-49	Hedmark	Arnestad Store
A4481	T16298	Male		>50	Sør-Trøndelag	Austrått
A4511	Ts4306	Female		20-35	Nordland	Rønvik Nedre
A4512	Ts4306	Male		20-35	Nordland	Rønvik Nedre
A4689	Ts5252	Female		35-49	Nordland	Vågehamn
A4691b	Ts5287	Male		22-35	Nordland	Hagbartholm
A4727	Ts5656	Male		40-50	Nordland	Stokke

A5195	Ts7659	Male		10-12	Nordland	Vikran Nordre
A5305	C35586	Male		35-49	Oppland	Skålgård Søndre
A5317	T20545	Female		15-17	Nordland	Prestgården
A7437 (I)	T20248		Not able	1-1.5	Nord-Trøndelag	Aunvoll
A7437 (Y)	T20248		Female	19-20	Nord-Trøndelag	Aunvoll
A7437 (O)	T20248		Female	35-49	Nord-Trøndelag	Aunvoll

Table S2: Overview of age development of teeth, from Scheid 2007 (58). Table S2 relate to Figure 3 and Figure 5.

Deciduous teeth		Beginning of hard tissue formation	Crown completion	Appearance	Root completion
	Maxilla				
	Central Incisor	In utero	4 month	7.5 month	1.5 year
	Lateral Incisor	In utero 4.5 month	5 month	9 month	2 years
	Canine	In utero 5 month	9 month	18 month	3.25 year
	1st molar	In utero 5 month	6 month	14 month	2.5 month
	2nd molar	In utero 6 month	11 month	24 month	3 year
Deciduous teeth					
	Mandibula				
	Central Incisor	In utero 4.5 month	3.5 month	6 month	1.5 year
	Lateral incisor	In utero 4.5 month	4 month	7 month	1.5 year
	Canine	In utero 5 month	9 month	16 month	3 year
	1st molar	In utero 5 month	10 month	12 month	2.25 year
	2nd molar	In utero 6 month	10 month	20 month	3 year
Permanent teeth					
	Maxilla				
	Central incisor	3-4 month	4-5 year	7-8 year	10 year
	Lateral incisor	10-12 month	4-5 year	8-9 year	11 year
	Canines	4-5 month	6-7 year	11-12 year	13-15 year
	1st premolar	1.5 – 1.75 year	5-6 year	10-11 year	12-13 year
	2nd premolar	2-2.25 year	6-7 year	10-12 year	12-14 year
	1st molar	Birth	2.5-3 year	6-7 year	9-10 year
	2nd molar	2.5 – 3 year	7-8 year	12-15 year	14-16 year
	3rd molar	7-9 year	12-16 year	17-21 year	18-25 year
Permanent teeth					
	Mandibula				

	Central incisor	3-4 month	4-5 year	6-7 year	9 year
	Lateral incisor	3-4 month	4-5 year	7-8 year	10 year
	Canine	4-5 month	6-7 year	9-10 year	12-14 year
	1st premolar	1.75-2 year	5-6 year	10-12 year	12-13 year
	2nd premolar	2.25-2.5 year	6-7 year	11-12 year	13-14 year
	1st molar	Birth	2.5-3 year	6-7 year	9-10 year
	2nd molar	2.5-3 year	7-8 year	11-13 year	14-15 year
	3rd molar	8-10 year	12-16 year	17-21 year	18-25 year

Table S3: Overview of human remains and age determination, both through ^{14}C and by the application of archaeological material, as well as overview of terrestrial and marine calibrated age determination as well as chronological dating through archaeological material. Abbreviation “A-number” means anthropological number, “M-number” means museum number. Table S3 relate to Figure S1,1A and Figure 4.

A-id	M-id	Sample id	2 sigma calibration	^{14}C mixed marine 95.4% probability	Archaeological dating	^{14}C interpretation
A253	C18558	Tra-14452	603-654 CE cal. Collagen	655-873 CE. collagen	8th to 11th Century	Death during the mid-7th to mid-9th centuries
A642	T5105	Tra-14913	892-992 CE cal. Carbon		8th to 9th Century	
A1502	C18035	Tra-15320	BCE 41-77 CE cal. Collagen	85-359 CE. collagen	4th to 5th Century	Death during the 4th century
A1517	C14690				10th to 11th Century	
A1520	C21852	Tra-14453	704-880 CE cal. Collagen	773-995 CE. collagen	8th to 11th Century	Death between the 9th and 10th century
A1522	C14554	Tra-15313	605-652 CE cal. Collagen	665-871 CE. collagen	8th to 9th Century	Death between the mid-8th and late 9th century
A1645	C21794	Tra-15316	435-567 CE cal. Collagen	442-651 CE. collagen	8th to 11th Century	Death between mid-7th and the 8th century
A2808(1)	C4438				8th to 11th Century	
A2808(2)	C4438				8th to 11th Century	
A2813	C17564	Tra-15315	703-878 CE cal. Collagen	774-1017 CE. Collagen	8th to 11th Century	Death between the 9th and early 11th century
A3697	C23941	Tra-14454	675-769 CE cal. Collagen	1024-1273 CE. Collagen	10th Century	Death during the 11th century
A3699	T13363	Tra-14919	BCE 1531-1447 cal. Collagen	BCE 1494-1229 Collagen	Not able	Death between BCE 1494 and 1229

A3705	T2327	Tra-14426	677-770 CE cal. Collagen	702-989 CE. Collagen	9th Century	Death during the 9th to 10th centuries
A3708	T9366	Tra-15321	656 -774 CE cal. Collagen	829-1121 CE. Collagen	10th Century	Death between th mid-10th and early 11th century
A3709	T12578	Tra-14423	656-774 CE cal. Collagen	666-892 CE. Collagen	9th to 10th Century	Death during the 9th century
A3777	C24243	Tra-14910	774-885 CE cal. Collagen	775-1016 CE Collagen	8th to 11th Century	Death during the mid - 9th to 10th century
A3778	C24297	Tra-14455	667-877 CE cal. Collagen		8th to 11th Century	
A4005	C25552	Tra-14457	775-887 CE cal. Collagen	776-1122 CE Collagen	8th to 11th Century	Death between the mid-9th and 11th century
A4006	C25720	Tra-14458	572-648 CE cal. Collagen	595-773 CE Collagen	10th Century	Death between the late 6th and 10th century
A4049	Ts3525	Tra-15312	600-646 CE cal. Collagen	695-987 CE Collagen	8th to 9th Century	Death between the late 8th and 9th Century
A4184	Ts3639	Tra-15323	646-676 CE cal. Collagen	763-995 CE Collagen	10th to 11th Century	Death between the 10th and 11th century
A4304	C26737				9th to 10th Century	
A4460	C27338	Tra-15322	774-886 CE cal. Collagen	774-1013 CE Collagen	10th to 11th Century	Death during the 10th century
A4481	T16298	Tra-14431	658-764 CE cal. Collagen	773-975 CE Collagen	10th to 11th Century	Death during the 10th century
A4511	Ts4306				10th to 11th Century	
A4512	Ts4306	Tra-14436	663-775 CE cal. Collagen	775-1025 CE Collagen	10th to 11th Century	Death between mid-10th and early 11th century
A4689	Ts5252	Tra-14438	990-1033 CE cal. Collagen	1181-1405 CE Collagen	8th to 10th Century	Death during the Late Viking Age
A4691b	Ts5287				8th to 11th Century	
A4727	Ts5656					
A5195	Ts7659	Tra-15314	435-579 CE cal. Collagen	648-883 CE Collagen	8th to 11th Century	Death during the 8th to late 9th centuries
A5305	C35586	Tra-15324	729-881 CE cal. Collagen	773-996 CE Collagen	9th to 11th Century	Death between the 9th and 10th century
A5317	T20545	Tra-14916	1025-1154 CE Cal. Carbonate		9th to 10th Century	
A7437 (I)	T20248	Tra-14441	1665-1927 CE Cal. Collagen	1677-1945 CE Collagen	Not able	Modern

A7437(Y)	T20248	Tra-14912	651-759 CE Cal. Collagen	664-875 CE Collagen	8th to 11th Century	Death between the late 8th and 9th century
A7437(O)	T20248	Tra-15329	649-673 CE Cal. Collagen	664-885 CE Collagen	Not able	Death between late 7th and late 9th century

Table S4 – overview over the individuals which display mobility through the oxygen isotpes ($\delta^{18}O$). Asterisk (*) indicating $\delta^{18}O$ results from Price and Naumann 2014 (51). Table S4 relate to Figure 1B, 3, 4, 6 and Figure 7.

A-id	M-id	$\delta^{18}O$	Tooth Bone Id	Develop years	$\delta^{18}O_{carb}$	Tooth id	Develop years	Areas of Mobility
A253	C18558	-4.9*	M2	2.5-8				
A642	T5105	-4.5*						
A1502	C18035	-5.94	M2	2.5-8				Borderline-regional mobility
A1517	C14690	-8.00	PM1	1-6				Probably local
A1520	C21852	-7.7*						
A1522	C14554	-5.18	M2	2.5-8				Southern Scandinavia, Northern Europe, UK, Ireland
A1645	C21794	-4.48	M1	0-3	-4.95	M3	7-16	Borderline-regional mobility
A2808(1)	C4438	-7.12	M1	0-3				Probably local
A2808(2)	C4438							
A2813	C17564	-6.82	M3	7-16				Probably local
A3697	C23941	-5.2*						
A3699	T13363							
A3705	T2327	-4.4*						
A3708	T9366	-5.26	M2	2.5-8				Southern Scandinavia, Northern Europe, UK, Ireland

A3709	T12578	-4.41	M2	2.5-8				Southern Scandinavia, Northern Europe, UK, Ireland, France
A3777	C24243	-7.62	PM1	1-6				Probably local
A3778	C24297							
A4005	C25552	-6.3*						
A4006	C25720	-4.3*						
A4049	Ts3525	-3.54	M1	0-3				S-W Spain, Portugal, Southern Italy, Northern Africa and Mediterranean
A4184	Ts3639	-5.63	M1 Max	0-3				Southern Scandinavia, Northern Europe, UK, Ireland
A4304	C26737	-7.13	M2	2.5-8				Probably local
A4460	C27338	-8.215	M2	2.5-8				Probably local
A4481	T16298	-6.22	M2	2.5-8				Borderline – regional mobility
A4511	Ts4306	-5.69	M1	0-3				Southern Scandinavia, Northern Europe, UK, Ireland
A4512	Ts4306	-5.07	M1	0-3				Southern Scandinavia, Northern Europe, UK, Ireland
A4689	Ts5252	-6.91	M1	0-3				Southern Scandinavia. Northern Europe, UK, Ireland, France
A4691b	Ts5287	-5.01	M1	0-3				Southern Scandinavia, Northern Europe, UK, Ireland, France

A5195	Ts7659	-4.73	M1	0-3				Southern Scandinavia, Northern Europe, UK, Ireland, France
A5305	C35586	-7.465	PM1	1-6				Probably local
A5317	T20545	-4.36	M1	0-3				Southern Scandinavia, Northern Europe, UK, Ireland, France
A7437(I)	T20248	-6.43	M2 deciduous	6 m. in utero-11 m.	-8.02			
A7437(Y)	T20248	-5.86	M3	7-16				Temperate Europe, UK, Ireland, Eastern France, Denmark, Germany
A7437(O)	T20248	-5.98	M1	0-3				Temperate Europe, UK, Ireland, Eastern France, Denmark, Germany

Table S5 – Overview of sword and axe grave goods referred to this study. The Asterix indicates that it is difficult to distinguish between which burial the weapon belongs to. Sources: Vike (2016), Androshchuk (2014) and Petersen (1919) (69,70,108). Table S5 relate to Figure 1B.

A-id	M-id	Farm	Sword type	Axe type	Distribution pattern
A642	T5105	Leines	C		Norway, Ireland, Sweden, Great Britain and France
A3708	T9366	Tommeidet	P?		Scandinavia, Iceland, Finland and Great Britain
A3777 or A3778*	C24243	Velo Nordre		M	Fitted with cylindrical brass, five findings in Norway, three similar axes found in the River Thames, eight in Gotland and one in Kaliningrad
A4049	Ts3525	Bø	C		Norway, Ireland, Sweden, Great Britain and France
A4304	C26737	Arnestad Lille	M		Singular findings in Scandinavia, Iceland, Great Britain, Ireland, Germany and France
A4511 and or A4512*	Ts4306	Rønvik Nedre	V/Y		V-type miniature sword-Y-type findings in Denmark, Finland, Iceland, Ireland, Rus and former Prussia

A4689	Ts5252	Vågehamn	C		Mass burial, C-type sword found in Norway, Ireland, Sweden, Great Britain and France
A5305	C35586	Skålgård Søndre	L		Norway, Sweden, Denmark, Iceland, Ireland, Belgium and Great Britain

Table S6 – Overview of diet results ($\delta^{13}C/\delta^{15}N$). Abbreviations: “Av” is average, “En” is enamel. Table S6 relate to Figure 2A and Figure 2B.

A-id	M-id	$\delta\ ^{13}C$ Av.	$\delta\ ^{15}C$ Av.	En. $\delta\ ^{13}C$ Av.	Tooth bone	$\delta\ ^{13}C$ Av.	$\delta\ ^{15}N$ Av.	En. $\delta\ ^{13}C$ Av.	Tooth/bone
A253	C18558	-19.48	12.55		M2				
A642	T5105								
A1502	C18035	-18.7	14.93	-14.71	Pars petrosa, M2				
A1517	C14690	-21.12	11.09	-15.11	Pars petrosa, PM1				
A1520	C21852								
A1522	C14554	-19.53	12.91	-14.43	Pars petrosa, M2				
A1645	C21794	-21.2	10.32	-16.92	Mandibula, M2			-14.29	M3
A2808 (1)	C4438	-20.67	10.16	-14.07	Mandibula, M1				
A2808 (2)	C4438	-20.98	9.65		Pars petrosa				
A2813	C17564	-20.2	11.73	-13.27	Pars petrosa, M3				
A3697	C23941								
A3699	T13363	-19.4	12.27	-14.92	Pars petrosa				
A3705	T2327	-20.42	13.1		M1				
A3708	T9366	-17.43	15.89	-13.41	Mandibula, M2				
A3709	T12578	-20.64	12.63	-14.17	M2	-20.53	11.3		Mandibula
A3777	C24243	-20.53	11.3		Mandibula, PM1				
A3778	C24297								
A4005	C25552								
A4006	C25720								
A4049	Ts3525	-17.35	16.4	-16.73	Mandibula, M1				
A4184	Ts3639	-18.58	15.49	-13.85	Maxilla, M1				
A4304	C26737			-14.51	M2				
A4460	C27338	-21.22	10.27	-15.025	Mandibula, M2				
A4481	T16298	-19.71	13.12	-15.79	M2	-21.39	11.52		Mandibula
A4511	Ts4306			-16.5	M1				
A4512	Ts4306	-18.74	15.3		M1				

A4689	Ts5252	-16.59	16.69	-12.15	M1	-16.37	16.23		Dental root M1
A4691B	Ts5287			-14.29	M1				
A4727	Ts5656								
A5195	Ts7659	-17.23	16.58	-14.85	Mandibula, M1				
A5305	C35586	-20.87	10.46	-14.565	Pm1	-21.2	9.81		Maxilla
A5317	T20545			-14.86	M1	-19.3	12.76		Mandibula
A7437(I)	T20248								
A7437 (Y)	T20248			-15.19	M3	-20.69	10.3		Mandibula
A7437 (O)	T20248	-20.19	10.73	-15.54	Mandibula, M1				

Table S7- table overview of C:N ratio. Abbrevations: “C” is Carbon, “N” is Nitrogen. Table S7 relate to Figure 2A and Figure 2B.

A-id	M-id	Sample Name	Sample number	C content % by weight	N Content % by weight	C:N ratio
A253	C18558	Tra14452	66	46	17	3.069325733
A642	T5105	Tra14421	3	35	13	3.287157011
A1502	C18035	Tra15320	11	44	16	3.173863454
A1517	C14690					
A1520	C21852	Tra14453	67	42	15	3.276166735
A1522	C14554	Tra15313	4	44	16	3.137052986
A1645	C21794	Tra15316	7	43	16	3.219823697
A2808 1	C4438					
A2808 2	C4438					
A2813	C17564	Tra15315	6	40	15	3.189106489
A3697	C23941	Tra14454	68	43	16	3.091350841
A3699	T13363	Tra14919	10	43	16	3.07506896
A3705	T2327	Tra14426	1	43	16	3.129917175
A3708	T9366	Tra15321	12	44	16	3.137041649
A3709	T12578	Tra14423	8	44	17	3.074104744
A3777	C24243	Tra14910	56	45	17	3.087475958
A3778	C24297					
A4005	C25552	Tra14457	70	45	17	3.09443578
A4006	C25720	Tra14458	71	45	17	3.060572776
A4049	Ts3525	tra15312	3	44	16	3.212535959
A4184	Ts3639	Tra15323	14	45	16	3.209801752
A4304	C26737					
A4460	C27338	Tra15322	13	42	16	3.163387562
A4481	T16298	tra14431	14	37	14	3.08652345
A4511	Ts4306					
A4512	Ts4306	tra14436	28	45	17	3.060043001

A4689	Ts5252	tra14438	32	44	17	3.09192939
A4691b	Ts5287					
A4727	Ts5656					
A5195	Ts7659	tra15314	5	40	15	3.156147344
A5305	C35586	tra15324	15	44	16	3.114091457
A5317	T20545					
A7437	T20248	tra14441	78	46	17	3.100870321
A7437	T20248	tra14912	3	38	14	3.100923853
A7437	T20248	tra15329	20	45	17	3.146574007

Table S8- table overview of collagen yield. Table S8 relate to Figure S1, 1A, Figure 2A and 2B.

Anthropological number	Museum number	Collagen yield
A253	C18558	11.42%
A642	T5105	84.26%
A1502	C18035	7.75%
A1517	C14690	1.71%
A1520	C21852	2.20%
A1522	C14554	2.39%
A1645	C21794	6.87%
A2808(1)	C4438	2.13%
A2808(2)	C4438	1.44%
A2813	C17564	4.04%
A3697	C23941	4.48%
A3699	T13363	1.37%
A3705	T2327	11.38
A3708	T9366	5.41%
A3709	T12578	3.85%
A3777	C24243	3.50%
A3778	C24297	37.70%
A4005	C25552	11.08%
A4006	C25720	11.65%
A4049	Ts3525	8.42%

A4184	Ts3639	4.41%
A4460	C27338	2.26%
A4481	T16298	7.77%
A4511	Ts4306	
A4512	Ts4306	8.06%
A4689	Ts5252	5.50%
A4691b	Ts5287	
A4727	Ts5656	
A5195	Ts7659	11.51%
A5305	C35586	3.32%
A5317	T20545	80.49%
A7437 (I)	T20248	4.18%
A7437 (Y)	T20248	5.63%
A7437 (O)	T20248	3.58%
